# Epidemiology of SARS-CoV-2 infection among staff and students in a cohort of English primary and secondary schools during 2020–2021

**DOI:** 10.1016/j.lanepe.2022.100471

**Published:** 2022-08-24

**Authors:** James R. Hargreaves, Sinéad M. Langan, William E. Oswald, Katherine E. Halliday, Joanna Sturgess, Jody Phelan, Patrick Nguipdop-Djomo, Benjamin Ford, Elizabeth Allen, Neisha Sundaram, Georgina Ireland, John Poh, Samreen Ijaz, Ian Diamond, Emma Rourke, Fiona Dawe, Alison Judd, Charlotte Warren-Gash, Taane G. Clark, Judith R. Glynn, W. John Edmunds, Chris Bonell, Punam Mangtani, Shamez N. Ladhani, COVID-19 Schools Infection Survey Study Group, Tanya Abramsky, Shazaad Ahmad, Felicity Aiano, Frances Baawuah, Urszula Bankiewicz, Sarah Batt, Joanne Beckmann, Ami Bhavsar, Bernadette Brent, Andrew Brent, Simon Brouwer, Kevin Brown, Richard Browne, Kevin Childs, Sarah Cook, Simon Cousens, Ieuan Day, Antonio Felton, Paul Fine, David Foster, Joanna Garstang, David Gates, Claire Grant, Bethany Griffiths-Tong, Claire Hele, Rowan Hemsi, Pete Jones, Helena Jordan, Adam Kucharski, Andrea Lacey, Rebecca Leeson, Ffion Lelii, Philip Lovely, Madeleine Lunskey, Chris McLanachan, James Munday, Ifeanyichukwu Okike, Kathleen O'Reilly, Penelope Parker, Annabel Powell, Sarah Proud, Mary Ramsay, Lee Rudd, Timothy Russell, Justin Shute, Nerissa Tilouche, Charmaine Virgin, Sian-Elin Wyatt, KELLY YEO

**Affiliations:** a Department of Public Health, Environments and Society, Faculty of Public Health and Policy, London School of Hygiene & Tropical Medicine, London, UK; b Department of Non-communicable Disease Epidemiology, Faculty of Epidemiology and Population Health, London School of Hygiene & Tropical Medicine, London, UK; c Department of Disease Control, Faculty of Infectious and Tropical Diseases, London School of Hygiene & Tropical Medicine, London, UK; d Department of Infectious Disease Epidemiology, Faculty of Epidemiology and Population Health, London School of Hygiene & Tropical Medicine, London, UK; e Department of Medical Statistics, Faculty of Epidemiology and Population Health, London School of Hygiene & Tropical Medicine, London, UK; f Department of Infection Biology, Faculty of Infectious and Tropical Diseases, London School of Hygiene & Tropical Medicine, London, UK; g Office for National Statistics, Government Buildings, Newport, UK; h Department of Global Health and Development, Faculty of Public Health and Policy, London School of Hygiene & Tropical Medicine, London, UK; i National Infection Service, UK Health Security Agency, London, UK; j Paediatric Infectious Diseases Research Group, St George's University of London, London, UK

**Keywords:** SARS-CoV-2, Schools, England

## Abstract

**Background:**

There remains uncertainty about the epidemiology of SARS-CoV-2 among school students and staff and the extent to which non-pharmaceutical-interventions reduce the risk of school settings.

**Methods:**

We conducted an open cohort study in a sample of 59 primary and 97 secondary schools in 15 English local authority areas that were implementing government guidance to schools open during the pandemic. We estimated SARS-CoV-2 infection prevalence among those attending school, antibody prevalence, and antibody negative to positive conversion rates in staff and students over the school year (November 2020–July 2021).

**Findings:**

22,585 staff and students participated. SARS-CoV-2 infection prevalence among those attending school was highest during the first two rounds of testing in the autumn term, ranging from 0.7% (95% CI 0.2, 1.2) among primary staff in November 2020 to 1.6% (95% CI 0.9, 2.3) among secondary staff in December 2020. Antibody conversion rates were highest in the autumn term. Infection patterns were similar between staff and students, and between primary and secondary schools. The prevalence of nucleoprotein antibodies increased over the year and was lower among students than staff. SARS-CoV-2 infection prevalence in the North-West region was lower among secondary students attending school on normal school days than the regional estimate for secondary school-age children.

**Interpretation:**

SARS-CoV-2 infection prevalence in staff and students attending school varied with local community infection rates. Non-pharmaceutical interventions intended to prevent infected individuals attending school may have partially reduced the prevalence of infection among those on the school site.

**Funding:**

UK Department of Health and Social Care.

**Research in context**

**Evidence before this study**

In January 2022, we searched PubMed using MeSH terms for “School” AND “SARS-CoV-2” and applying filters for “reviews” AND “Systematic reviews”. The search identified 58 papers, of which 8 were systematic reviews of the epidemiology of SARS-CoV-2 in school populations, and/or implementation and effectiveness of school measures to control COVID-19. Four reviews summarised evidence on transmission and epidemiological studies in schools and children. One examined evidence linking school closures with impacts on community transmission, one examined infection risks for teachers, and two reviews summarised evidence and guidelines related to measures implemented within schools. The reviews documented measures recommended for implementation by schools in response to the SARS-CoV-2 pandemic and a need for stronger evidence on the role of schools in transmission.

**Added value of this study**

The COVID-19 Schools Infection Survey in England is one of the largest and most comprehensive longitudinal research studies undertaken globally in primary and secondary schools to date during the COVID-19 pandemic (156 schools, 22,585 participants). The study involved: repeated in-school collection of biological samples for current infection and antibodies to SARS-CoV-2 to capture past infection; online questionnaires; and linkage to routine data sources. We used these data to measure SARS-CoV-2 infection rates among staff and students over the school year, in the context of guidance on the implementation of policy measures to limit school transmission, including partial and full school closures and so-called “school-gate” efforts to limit the extent to which infected individuals were present on the school campus.

**Implications of all the available evidence**

In the context of high levels of ongoing community-based transmission during September-December 2020, the overall prevalence of current SARS-CoV-2 infection among staff and students attending school on normal school days was 0.7–1.6%. Over the school year, antibody prevalence rose among staff and students. During April–July 2021, a mass asymptomatic testing programme had been introduced for school staff and secondary school students, in addition to a range of existing measures. In both the autumn and summer terms, the prevalence of current infection among secondary school students attending school was lower than the prevalence among secondary-school age children measured in the community in the same region. Overall, the study findings suggest that the epidemiology of SARS-CoV-2 among school populations varied with community transmission, and that “school gate” control measures in place during 2020–21 may have partially reduced the risk of those with prevalent infection attending school.

Alt-text: Unlabelled box

## Introduction

The “severe acute respiratory syndrome coronavirus 2” (SARS-CoV-2) outbreak was declared a global pandemic by the World Health Organisation in March 2020.^1^^,^^2^ By this date, population restriction measures, including school closures, were being implemented globally to reduce transmission.^3^, ^4^, ^5^, ^6^ While children under 18 years are less likely to develop severe COVID-19 than adults,^7^ experience with influenza pandemics had shown that school closures reduced the size of outbreaks.^8^ The role of children and schools in the transmission of SARS-CoV-2 was unclear.^9^^,^^10^ Following the initial school closures, many countries, including the UK, issued guidance on mitigation measures in educational settings to limit transmission when schools reopened after national lockdown.

The first wave of SARS-CoV-2 infections in England began in March 2020 and led to a national lockdown on 23 March 2020, including school closures until the end of May 2020 for most children. Subsequently, there was a period of low incidence of SARS-CoV-2 infections, hospitalisations, and deaths during the summer. The Department for Education (DfE) in England updated its guidance in July 2020 to help schools reopening for in-person teaching for all students at the start of the 2020/21 academic year (Table 1). Additional mitigation measures were introduced during the year. While many “within-school” measures were principally aimed at reducing the risk of in-school transmission from infected individuals to others, some “school gate measures”, such as the roll-out of mass asymptomatic testing for staff and secondary school children from March 2021, were intended to limit infected persons from entering the school premises.

**Table 1:** Summary of DfE guidance to schools for Autumn term 2020 and major additions during the school year.^11^

Educational settings should: • Thoroughly review health and safety risk assessments. • Draw up plans that address the risks identified using the system of controls. Essential measures include: • Minimise contact with individuals who are unwell by ensuring that those who have coronavirus (COVID-19) symptoms, or who have someone in their household who does, do not attend school.^a^ • Use of face coverings, where recommended. • Clean hands thoroughly more often than usual. • Ensure good respiratory hygiene by promoting the ‘catch it, bin it, kill it’ approach. • Introduce enhanced cleaning, including cleaning frequently touched surfaces often, using standard products such as detergents and bleach. • Active engagement with NHS Test and Trace, which in the main includes (i) advising the school community to book a PCR test and self-isolate, if symptomatic, and provide details of close contacts, if asked, and (ii) for the school to report cases to the government.^a^ • Managing confirmed cases amongst the school community and containing outbreaks by following the advice of local health protection teams. Consider: • Formal consideration of how to reduce contacts and maximise distancing between those in the setting and, wherever possible, minimise the potential for contamination as much as is reasonably practicable. • Use of appropriate PPE, where necessary. Reducing contact will depend on the setting's individual circumstances and will include, as much as possible: • Grouping children and young people together in contact bubbles. • Self-isolating if exposed within bubbles.^a^ • Avoiding contact between groups. • Arranging classrooms with forward facing desks. • Staff maintaining distance from pupils and other staff as much as possible. • For younger children the focus will likely be on keeping groups separate, while for older children it would be on distancing. Major additions during the study period: • Launch of rapid asymptomatic testing using lateral flow devices (LFDs) in secondary schools and colleges in a phased approach beginning in January 2021, for staff and students in school during the national lockdown, and three on-site tests for students prior to return to school for all students on 8 March 2021. Biweekly testing at home thereafter for all secondary school staff and students and primary school staff.^a^ • Face coverings in classrooms for students and adults in secondary schools (March 2021). Previously they were recommended in corridors and communal areas where social distancing could not easily be maintained. At primary schools, a recommendation for face coverings for adults in communal areas remained, with no additional face covering requirements in the classroom or for students. • Emphasis on ventilation of occupied spaces.

a “School gate” measures principally intended to limit the chance of infected individuals being present on the school site.

The aim of the COVID-19 Schools Infection Survey (SIS) was a multi-objective study to assess the role of schools in SARS-CoV-2 transmission in England during the school year 2020–2021. The study ran from November 2020 to July 2021. In the analysis we present here, we estimated SARS-CoV-2 infection prevalence among those attending school on normal school days, antibody prevalence, and antibody conversion rates among staff and students in participating schools. We explored the relationship between community case rates, periods of school opening and community social distancing over the year. Furthermore, in North-West England, the region with highest SARS-CoV-2 infection prevalence at the start of the school year 2020–2021, we explored whether SARS-CoV-2 infection prevalence among children sampled while attending school differed from the estimated prevalence of SARS-CoV-2 infection among all children of the same age in the region.

## Methods

### Study design and context

SIS was a longitudinal, population-based observational study in a cohort of students and staff from selected schools in 15 local authorities (LAs) across England with stratified sampling based on case rates in September 2020.^12^ The study was designed to run through the school year 2020–2021 with open enrolment and plans for six rounds (two per term) of school- and home-based (from round 2) sample collection.

The sampling design and data-collection methods of the study have been described previously.^12^ Briefly, during the autumn term of the school year 2020–2021, we set out to recruit approximately 50 primary and 100 secondary schools from 15 LAs in England to participate in a year-long cohort study. To facilitate transmission studies, we selected LAs to over-represent locations with a high burden of community COVID-19 cases at the start of the 2020–2021 school year. We selected ten LAs randomly from among those with case rates in the top 20% nationally in September 2020, and a further five LAs were randomly selected from the remainder of LAs nationally. The study did not seek to estimate outcomes that were intended to be generalizable at national level, but rather to report on outcomes over time within selected schools in regions with high and low community SARS-CoV-2 infection prevalence.

**Figure 1:**
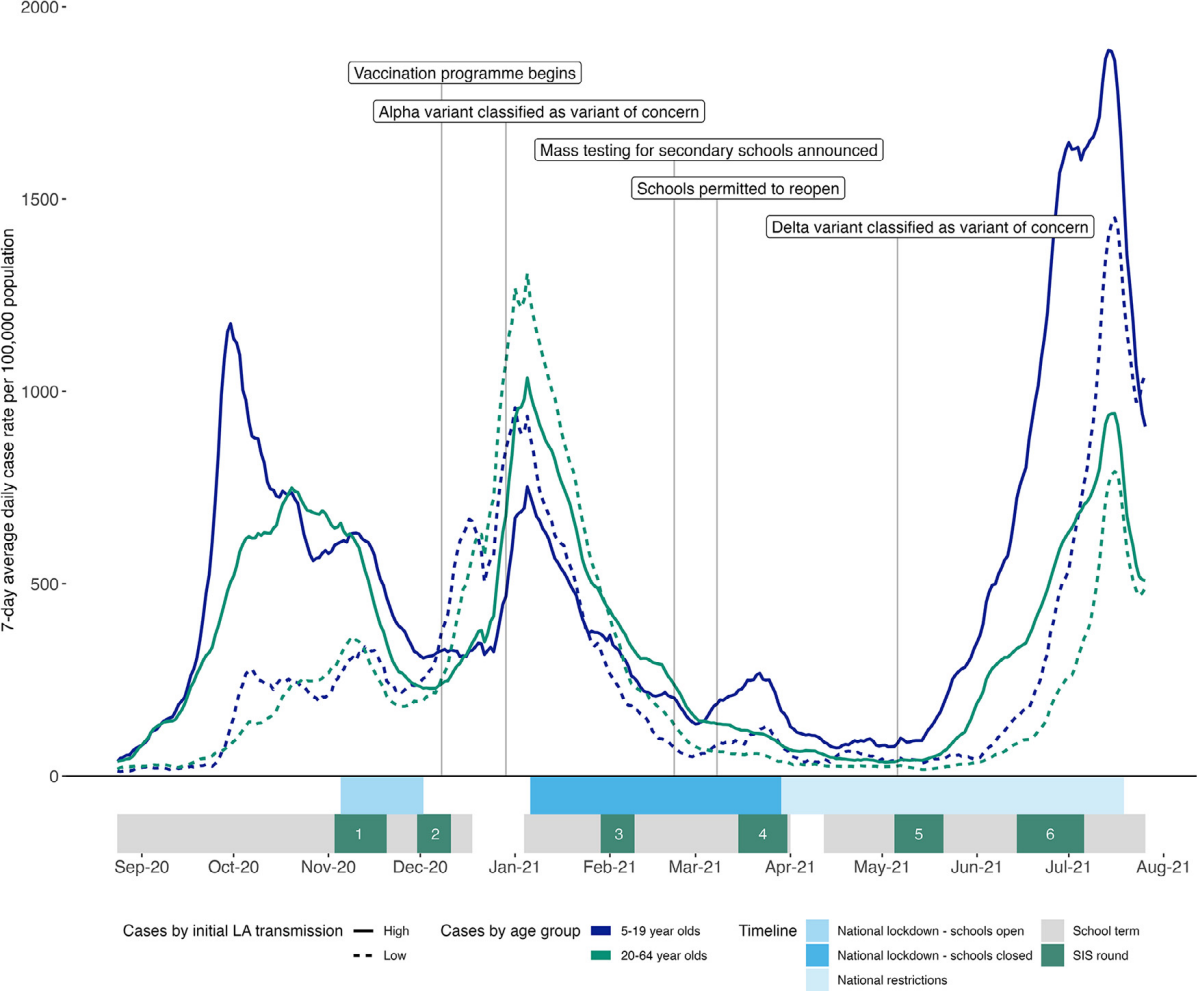
Schools Infection Survey context, September 2020–July 2021, including population case rates over time in study local areas by initial transmission status and age groups, school term dates, national lockdowns, school closures, and other major relevant public health events.

Figure 1 illustrates the broader epidemiological context within which SIS was conducted. Rolling seven-day mean case numbers from community testing in sampled LAs are shown, stratified by SARS-Cov-2 infection prevalence from September 2–8, 2020 (https://coronavirus.data.gov.uk/details/cases). SIS testing rounds are shown, as well as school closures and national community “lockdowns” in response to the pandemic.^12^ Although the initial high prevalence LAs had the highest prevalence in Autumn 2020, this pattern changed with the Alpha variant in early 2021. Schools were closed to most students from 06 January to 08 March 2021 and remained open when the Delta variant emerged in England from April 2021.

### Study procedures

There was a rolling recruitment process for schools and participants (people who provided at least one sample) within those schools from October 2020 to May 2021, although not all schools participated in all the rounds.^12^ All students from 4 years of age and all staff in primary schools were eligible to join the study from the start (November 2020). For secondary students, in the first term (November-December 2020), only those from two systematically selected consecutive year-groups were eligible in any one school: years 7 and 8 (ages 11–13); 9 and 10 (ages 13–15); or years 12 and 13 (ages 16–18) were randomly selected to participate in each school. In January 2021, the offer of enrolment was expanded to all students in secondary schools, except those from year 11 who, following guidance from the Department for Education, were not invited to participate throughout the year to limit disruption to learning during their final examination year. The sample size and design for the study were limited by pragmatic and logistical considerations as described previously.^12^

Laboratory methods are described in detail elsewhere.^12^ In brief, we collected nasal swabs for real-time polymerase chain reaction (“real time-PCR”) testing for current SARS-CoV-2 infection, oral fluid swabs for antibody testing among students, and self-sampled capillary blood samples for antibody testing among staff, during in-school visits. We considered oral fluid collection a more feasible method for large scale collection of samples from students than capillary blood sampling. Two visits were undertaken to schools in each term, except that in-school sampling was cancelled in January 2021 (round 3) during England's third national lockdown during the alpha variant wave. In addition, postal collection of home sampling kits for antibody testing was introduced from round 2. Anyone enrolled in the study but absent from school on the visit day was invited to return a test sample taken at home. We invited participants to complete baseline and follow-up questionnaires through an online platform.

Nasal swabs were sent to a national Lighthouse testing laboratory (Glasgow) assay.^13^^,^^14^ Oral fluid swabs were sent to Public Health England Colindale (now UK Health Security Agency) for detection of antibodies against the SARS-CoV-2 Nucleoprotein (NP) using an Immunoglobulin G (IgG)-capture-based enzyme immunoassay (EIA).^15^ Capillary blood samples taken from staff were tested with a commercial immunoassay for Nucleoprotein (NP) antibodies against SARS-CoV-2 (Roche cobas® Elecsys Anti-SARS-CoV-2 assay). Both antibody assays used in the study detect antibodies deriving from natural infection, but not those derived through vaccination.^16^

### Statistical analysis

We estimated crude infection prevalence and antibody prevalence per sampling round by school type (primary and secondary) and participant type (staff and students), and by area. We estimated confidence intervals based on robust standard errors to account for clustering within schools and LAs. When calculating antibody prevalence among students, we made an adjustment based on the sensitivity (80%) and specificity (99%) of the oral fluid assay used,^17^ using the formula: *p*=(*q*+specificity−1)/(sensitivity+specificity−1), where *p* is the adjusted proportion positive, and *q* is the observed proportion positive.^18^ We estimated rates of conversion from antibody negative to positive (indicating incident infections) between testing rounds as the number of antibody conversions per total person-time in weeks, including individuals who were antibody negative at the start of the period and had a valid antibody result at the following round. Reported school visit dates were used to determine follow-up time if available and, if not, the midpoint date of each round was used. Jackknife confidence intervals were estimated to account for clustering by school. When calculating incidence rates based on antibody conversion, individuals were not included in the analysis of subsequent rounds after a round with a positive antibody result. We did not seek to estimate rates of conversion from antibody positive to negative, and we did not directly study antibody waning in this study. Estimates of the prevalence of key outcomes at LA level are presented in supplementary information (Tables S5a-S6d).

The large number of schools from LAs in the North-West region (Manchester, Salford, Warrington, Liverpool, Knowlsey, Lancashire) in SIS allowed us, in a post-hoc analysis, to compare the prevalence of current infection in primary and secondary school students attending school with published estimates of community prevalence from the same time periods and region from the ONS Coronavirus (COVID-19) Infection Survey (CIS) among children aged 2–11 and 12–18 years.^19^ Comparison was only possible for rounds 1, 2 and 6 owing to the small number of current infections detected in rounds 3, 4, and 5 in SIS.

### Role of the funding source

This report is independent research funded by the Department of Health and Social Care (COVID 19- NTP 2.0, School Infection Study). The views expressed in this publication are those of the author(s) and not necessarily those of the NHS or the Department of Health and Social Care.

## Results

The number of participating primary schools increased from 45 in round 1 to 57 in rounds 4–6 (Table 2). In secondary schools, 62 participated in round 1, with the number rising to 80, 86, 91, 89 and 86 in subsequent rounds (Figures S1 and S2).

**Table 2:** Eligibility, participation, and response rates for primary-school staff, primary-school students, secondary-school staff, and secondary-school students over 6 SIS rounds, 2020–2021.

			Staff			Student		
School type	Round	Schools	Participating/eligible (%)	Valid swab test/participating (%)	Valid antibody test/participating (%)	Participating/eligible (%)	Valid swab test/participating (%)	Valid antibody test/participating (%)
**Primary**	1	45	1159/2485 (46.6)	1121/1159 (96.7)	1034/1159 (89.2)	2164/18066 (12.0)	2090/2164 (96.6)	2149/2164 (99.3)
	2	43	1189/2438 (48.8)	1082/1189 (91.0)	1013/1189 (85.2)	2434/17576 (13.8)	2153/2434 (88.5)	2414/2434 (99.2)
	3^a^	42	72/575 (12.5)	-	66/72 (91.7)	193/742 (26.0)	-	191/193 (99.0)
	4	57	1441/3000 (48.0)	1376/1441 (95.5)	1138/1441 (79.0)	3707/21520 (17.2)	3653/3707 (98.5)	3628/3707 (97.9)
	5^a^	57	1417/2984 (47.5)	1351/1417 (95.3)	1051/1417 (74.2)	4091/21474 (19.1)	4037/4091 (98.7)	519/4091 (12.7)
	6	57	1247/2959 (42.1)	1123/1247 (90.1)	842/1247 (67.5)	3932/21450 (18.3)	3678/3932 (93.5)	3850/3932 (97.9)
**Secondary**	1	62	3165/7583 (41.7)	3042/3165 (96.1)	2773/3165 (87.6)	3053/22478 (13.6)	2965/3053 (97.1)	3032/3053 (99.3)
	2	80	3988/9943 (40.1)	3618/3988 (90.7)	3291/3988 (82.5)	4570/29198 (15.7)	4006/4570 (87.7)	4461/4570 (97.6)
	3^a^	86	495/2504 (19.8)	-	467/495 (94.3)	1877/4107 (45.7)	-	1850/1877 (98.6)
	4	91	3033/11204 (27.1)	2933/3033 (96.7)	2214/3033 (73.0)	7482/53832 (13.9)	7347/7482 (98.2)	7308/7482 (97.7)
	5^a^	89	2784/10839 (25.7)	2644/2784 (95.0)	1985/2784 (71.3)	8265/52963 (15.6)	8062/8265 (97.5)	1343/8265 (16.2)
	6	86	2290/10374 (22.1)	2164/2290 (94.5)	1594/2290 (69.6)	7307/51707 (14.1)	6866/7307 (94.0)	7203/7307 (98.6)

a Round 3 was implemented via home testing as school visits were cancelled due to lockdown. Eligible individuals were contacted and sent home test kits. Eligible individuals were all individuals who had either (a) enrolled before 28th January 2021 but had not participated in Rounds 1 or 2, or (b) had participated in Rounds 1 or 2 but did not have a valid antibody test. Owing to resource constraints, at Round 5 not all samples collected from students were processed in the laboratory. Samples were processed from students who had only one other sample collected in another round.

Participation among primary school students increased from 12.9% across rounds 1 and 2, to 18.2% across rounds 4 to 6 (Table 2). Participation for secondary school students remained relatively stable between 13.6% and 15.7% across survey rounds. This pattern remained unchanged after 55 of the enrolled secondary schools expanded enrolment to additional year-groups beyond the original two school year groups sampled in autumn term 2020. While primary school staff participation was consistent across rounds, staff participation in secondary schools was 40.8% across rounds 1 and 2, falling to 25.0% across rounds 4 to 6. In both primary and secondary schools, the percentage of participating staff providing finger-prick blood samples for antibody testing was lower at every round than the percentage with swab test results, declining from 89.2% to 67.5% between rounds 1 and 6. Swab testing remained high with >90% of participating staff having valid swabs at each round (Table 2). No declines over time were seen in the proportion (97%) of participating students providing oral fluid samples for antibody testing.

Of the 22,585 participants in the study, 62.8% were in LAs with high community prevalence at the start of school year and most (92.7%) were from urban settings (Table 3). Staff were mostly female, especially in primary schools (90.2%), and >90% were White. Primary and secondary school students had similar proportions of males and females. The majority were of White ethnicity; 11.6% of primary school students and 6.1% of secondary school students were of Asian/Asian British ethnicity and 2.5% of primary school students and 2.2% of secondary school students were of Black/African/Caribbean/Black British ethnicity. By round six, 86.5% of staff were vaccinated (Table 3). Participant characteristics were similar in each round (Tables S1a-S1d).

**Table 3:** Sociodemographic characteristics of primary-school staff, primary-school students, secondary-school staff, and secondary-school students who participated in any SIS round, 2020–2021.

		Primary Staff	Primary students	Secondary staff	Secondary students	All
**Overall**	–	1891 (100)	4654 (100)	5852 (100)	10,188 (100)	22585 (100)
**Transmission Sep-2020**	Low	647 (34.2)	1523 (32.7)	2126 (36.3)	4114 (40.4)	8410 (37.2)
	High	1244 (65.8)	3131 (67.3)	3726 (63.7)	6074 (59.6)	14175 (62.8)
**School rural/urban**	Rural	177 (9.4)	594 (12.8)	322 (5.5)	545 (5.3)	1638 (7.3)
	Urban city and town	739 (39.1)	2001 (43.0)	2849 (48.7)	6056 (59.4)	11645 (51.6)
	Urban conurbation	975 (51.6)	2059 (44.2)	2681 (45.8)	3587 (35.2)	9302 (41.2)
**Age group (years)**	<5 years	0 (0.0)	346 (7.5)	0 (0.0)	0 (0.0)	346 (1.5)
	5-9 years	0 (0.0)	3269 (71.1)	0 (0.0)	0 (0.0)	3269 (14.6)
	10-14 years	0 (0.0)	981 (21.3)	0 (0.0)	8325 (82.1)	9306 (41.6)
	15+ years	0 (0.0)	0 (0.0)	0 (0.0)	1814 (17.9)	1814 (8.1)
	<35 years	502 (26.9)	0 (0.0)	1879 (32.5)	0 (0.0)	2381 (10.6)
	35-44 years	495 (26.5)	0 (0.0)	1712 (29.6)	0 (0.0)	2207 (9.9)
	45-54 years	559 (29.9)	0 (0.0)	1383 (23.9)	0 (0.0)	1942 (8.7)
	55+ years	312 (16.7)	0 (0.0)	811 (14.0)	0 (0.0)	1123 (5.0)
	Data not available	23	58	67	49	197
**Gender**	Male	183 (9.8)	2322 (50.6)	1532 (26.5)	5049 (49.9)	9086 (40.6)
	Female	1683 (90.2)	2270 (49.4)	4244 (73.5)	5076 (50.1)	13273 (59.4)
	Data not available	25	62	76	63	226
**Ethnicity**	Asian/Asian British	96 (5.2)	528 (11.6)	222 (3.9)	617 (6.1)	1463 (6.6)
	Black/African/Caribbean/Black British	12 (0.6)	114 (2.5)	56 (1.0)	222 (2.2)	404 (1.8)
	Mixed/Multiple ethnic groups	20 (1.1)	264 (5.8)	118 (2.1)	446 (4.4)	848 (3.8)
	Other ethnic group	6 (0.3)	51 (1.1)	30 (0.5)	94 (0.9)	181 (0.8)
	White	1726 (92.8)	3596 (79.0)	5327 (92.6)	8698 (86.3)	19347 (87.0)
	Data not available	31	101	99	111	342
**IMD 2019 quintiles**	1	425 (23.0)	1257 (27.5)	924 (16.2)	2248 (22.4)	4854 (21.9)
	2	369 (19.9)	985 (21.6)	1213 (21.2)	2081 (20.7)	4648 (21.0)
	3	315 (17.0)	715 (15.7)	1077 (18.8)	1653 (16.4)	3760 (16.9)
	4	383 (20.7)	877 (19.2)	1259 (22.0)	1924 (19.1)	4443 (20.0)
	5	358 (19.4)	731 (16.0)	1244 (21.8)	2148 (21.4)	4481 (20.2)
	Data not available	41	89	135	134	399
**Key Stage (year group)**	KS-1	–	1848 (40.3)	–	0 (0.0)	1848 (12.6)
	KS-2	–	2743 (59.7)	–	0 (0.0)	2743 (18.7)
	KS-3	–	0 (0.0)	–	7284 (72.0)	7284 (49.5)
	KS-4	–	0 (0.0)	–	1658 (16.4)	1658 (11.3)
	KS-5	–	0 (0.0)	–	1168 (11.6)	1168 (7.9)
	Data not available	–	63	–	78	141
**Job group (staff only)**	Senior leader	172 (9.3)	–	453 (7.9)	–	625 (8.2)
	Middle leader	115 (6.2)	–	1180 (20.5)	–	1295 (17.0)
	Teacher	491 (26.6)	–	2032 (35.3)	–	2523 (33.2)
	TA/Special Ed	598 (32.4)	–	528 (9.2)	–	1126 (14.8)
	Admin/Pastoral	187 (10.1)	–	938 (16.3)	–	1125 (14.8)
	Cater/Clean/Maintenance	147 (8.0)	–	226 (3.9)	–	373 (4.9)
	Other	133 (7.2)	–	405 (7.0)	–	538 (7.1)
	Data not available	48	–	90	–	138
**1 or more vaccine doses by round 6**	No	266 (14.1)	–	779 (13.3)	–	1045 (13.5)
	Yes	1625 (85.9)	–	5073 (86.7)	–	6698 (86.5)

IMD = Index of multiple deprivation.

Key stage is a stage of the state educational system in England. Primary school includes KS-1 (school years 1 and 2, ages 5–7) and KS-2 (years 3–6, ages 7–11), while secondary school include KS-3 (years 7–9, age 11–14), KS-4 (10–11, ages 14–16) and KS-5 (years 12–13, ages 16–18).

TA= Teaching assistant.

**Figure 2:**
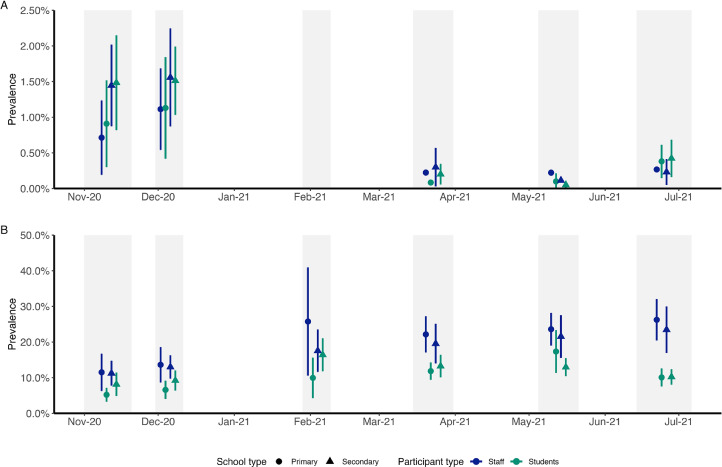
(A-B): (A) Current infection prevalence and (B) nucleoprotein antibody, indicating prior infection irrespective of vaccination status, prevalence (student estimates adjusted for sensitivity and specificity) at each round by school and participant type during 6 SIS rounds, 2020–2021, accounting for clustering by school and local area. Grey bars indicate timings of rounds 1–6. Current infection markers without confidence intervals (see rounds 4, 5 and 6 in panel A) indicate positive case counts below 3 that could not be published and that have been displayed as a maximum prevalence value calculated from 3 positive cases per group.

Figure 2 shows estimates of infection and antibody prevalence during the school year and by participant type. Infection and antibody prevalence varied considerably between and within LAs (estimates by round and participant type are provided in Supplementary Information Tables S2-S4). The prevalence of current SARS-CoV-2 infection among those attending school was highest during the first two rounds of testing in the autumn term (Figure 2a), with estimates ranging from 0.7% (95% CI 0.2, 1.2) among primary school staff in round 1 to 1.6% (95% CI 0.9, 2.2) among secondary school staff in round 2 (Table S2). Comparing staff and students, and primary and secondary schools, confidence intervals overlapped for all rounds. At rounds 1, 2 and 4, prevalence was non-significantly higher among secondary school staff and students than among primary school staff and students. The lowest prevalence of infection was seen in rounds 4 and 5.

The prevalence of nucleoprotein antibodies in oral fluid samples collected from students (Figure 2b), was consistently lower than the prevalence in staff, measured using capillary blood samples. Among primary and secondary staff, antibody prevalence based on capillary blood samples rose during the school year rose from 11.5% and 11.3%, respectively, in round 1, to 26.3% and 23.5% in round 6. Among students, antibody prevalence was non-significantly higher among secondary than primary school students in rounds 1, 2, and 4, but very slightly lower in round 6 among secondary students (Table S3). Student antibody prevalence rose from rounds 1 to 4 from 5.3–9.1% in round 1 to 13.7–15.5% in round 4 but was lower at round 6 than round 4. At round 6 antibody prevalence was higher among staff than students in both primary and secondary schools with no overlap in confidence intervals.

**Figure 3:**
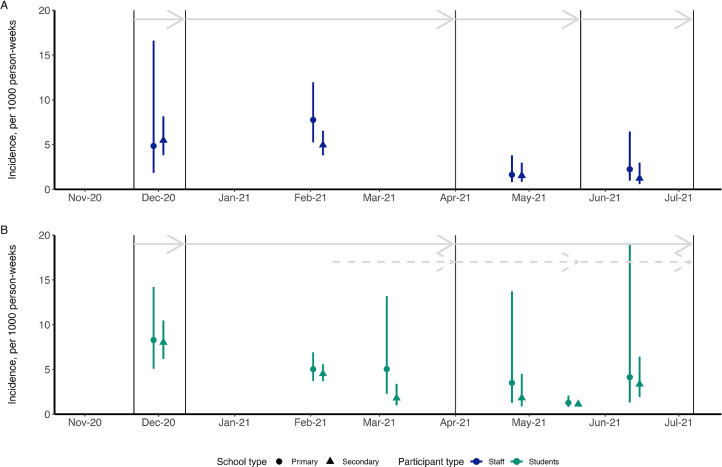
(A-B): Antibody conversion incidence rates during follow-up periods between SIS survey rounds by school for staff (A) and students (B), 2020–2021. Vertical bars and grey arrows indicate approximate follow-up periods. Grey dashed arrows indicate additional follow-up periods for subset of students with antibody testing results for rounds 3 and 5. Staff numbers for follow-up from round 2 to 3 and 3 to 4 and student numbers for follow-up from round 2 to 3 were too low to present or estimate confidence intervals. Jackknife confidence intervals are presented to account for clustering by school.

Figure 3 shows antibody conversion rates during the school year by school and participant type. The rate of antibody conversion was highest among primary students between round 1 and 2 (8.3 per 1000 person-weeks, 95% CI 5.1–14.2) and lowest among secondary school students between rounds 4 and 6 (1.1 per 1000 person-weeks, 95% CI 0.8–1.5) (Table S4).

Table 4 shows the comparison of current SARS-CoV-2 infection prevalence among those attending schools sampled from the LAs in the North-West region of England at three time-points (rounds 1, 2 and 6) with estimates of the community prevalence among 2–11 year-olds and 12–16 year-olds included in CIS in the same region at comparable time-periods. The estimates of prevalence of current infection among those sampled in primary school were lower than the regional prevalence estimates among 2–11 year-olds (proxy for primary-school children) at all rounds, though confidence intervals overlapped. Among secondary-school students in round 1, prevalence was 55% lower than among 12–16 year-olds enrolled in CIS, with no overlap in confidence intervals. In the summer term (round 6), the prevalence of infection among secondary school children was 75% lower with no overlap in confidence intervals.

**Table 4:** Current SARS-CoV-2 infection prevalence among SIS participant students sampled in-school and community infection among children in the North-West region of England during SIS rounds 1, 2 and 6, 2020–2021.

Period	Age Band	CIS Testing positive % (95% CI)	SIS Testing positive % (95% CI)
08 November to 21 November 2020 (CIS)/Round 1 (SIS)	Age 2 - Age 11 (CIS) /Primary (SIS)	2.09 (1.47, 2.89)	1.00 (0.48, 1.84)
22 November to 05 December 2020 (CIS)/Round 2 (SIS)		1.28 (0.79, 1.94)	0.99 (0.45, 1.86)
13 June to 26 June 2021 (CIS)/Round 6 (SIS)		0.79 (0.31, 1.62)	0.71 (0.26, 1.53)
08 November to 21 November 2020 (CIS)/Round 1 (SIS)	Age 12 - Age 16 (CIS) /Secondary (SIS)	4.48 (3.27, 5.97)	2.03 (1.19, 3.23)
22 November to 05 December 2020 (CIS)/Round 2 (SIS)		2.37 (1.54, 3.49)	1.27 (0.73, 2.05)
13 June to 26 June 2021 (CIS)/Round 6 (SIS)		2.05 (1.04, 3.63)	0.51 (0.25, 0.91)

CIS = Coronavirus (COVID-19) Infection Survey^20^; SIS = Schools Infection Survey. Data from the North West region in the Schools Infection Survey relates to six Local Authorities (Knowsley, Lancashire, Liverpool, Manchester, Salford, Warrington) and data from the COVID-19 Infection Survey relates to the whole of the North West region. Dates refer to 14-day periods used from the COVID-19 Infection Survey that relate to the period where the majority of tests were collected in each Schools Infection Survey round. CIS estimates are weighted^21^ SIS figures are unweighted.

## Discussion

During the autumn 2020 school term in England, within our sample of schools, approximately 1% of staff and students were infected with SARS-CoV-2 when tested on-site on normal school days. This in-school prevalence was much lower by summer 2021. Over the year, patterns of infection among those on school premises were similar between staff and students and between primary and secondary school settings. In the North-West region of England, where we were able to make comparisons with the ONS Coronavirus (COVID-19) Infection Survey, infection prevalence among secondary school students in school was 55% lower in round 1 and 75% lower in round 6 than the estimated overall prevalence of infection among secondary school age students in the region, with no overlap in confidence intervals.

The prevalence of SARS-CoV-2 nucleoprotein antibodies (consistent with previous infection) among school staff rose from about 10% in November 2020 to 25% by July 2021. Patterns were similar in primary and secondary schools. Among students, antibody prevalence was lower than for staff, even after correction for test characteristics, peaking at 10% and remaining stable, with some suggestion of a fall, over the summer term. Antibody conversion rates between sampling rounds among both staff and students, which should approximate the total incidence of infection irrespective of where infection was acquired, varied over time in a manner similar to community case rates.

Our findings confirm the potential for school-based transmission of SARS-CoV-2, especially where community levels of infection are high. During the first term of the school year, as community infection rates rose, and despite prevailing advice to quarantine at home if infected or with symptoms, we found evidence of (presumably asymptomatic) infected individuals being present on both primary and secondary school premises on routine school days. At the time, there were no recommendations for regular home testing for SARS-CoV-2 for students or staff.

By the summer term 2021, most school staff alongside the rest of the adult population had been vaccinated and had access to regular rapid home testing for SARS-CoV-2. Our data also suggest a higher SARS-CoV-2 prevalence among children in the community than among children attending school in the North-West, especially in secondary school students. This pattern is consistent with the idea that control measures implemented at “the school gate” (Table 1) may have had some positive effects in limiting the proportion of infected individuals attending school among both staff and students. Of note, by the final round of the study both exclusion of cases and their class contact “bubbles”, along with a programme of twice-weekly testing of asymptomatic staff and students were recommended across secondary schools. In primary schools, testing was only recommended for staff. The summer term coincided with the lowest prevalence estimates of infection found on site and lower prevalence among those attending school on normal school days than the estimated community prevalence in secondary age children. A consequence of these measures, however, was a high rate of school-absenteeism because of home isolation of cases and the large number of contacts in the class “bubble” per case during the delta variant surge in June/July 2021. In addition, antibody prevalence levels did not suggest that school staff were at greater cumulative risk of infection than other working age adults.^22^, ^23^, ^24^

SIS was a large, rapidly launched, national-scale study conducted across 15 different geographic areas, with systematic sampling of staff and students and administration of standardised questionnaires. The study was not designed to be nationally representative. Rather, the aim was to focus on understanding the risk of transmission in schools through oversampling of areas with high community infection prevalence at the start of the school year in September 2020. The design allowed comparisons to be made between staff and students, and between primary and secondary settings, and exploratory comparisons with community background rates of infection. Nested studies explored the feasibility and acceptability of school implementation of control measures, risk factors for infection among staff and students and mathematical modelling to estimate the relative importance of school and community transmission and the impact of school control measures. In this analysis we did not seek to investigate transmission risk within the school setting, although a previous analysis has investigated secondary attack rates using some data collected in SIS schools.^25^

The main limitation of the study relates to relatively low response rates, particularly among students, despite a range of efforts including compensating participating schools. While our non-response rates were similar to other national-scale studies launched in the UK during the pandemic,^26^^,^^27^ participants who volunteered to take part may have differed in important ways from those who declined. Hence, we may have incorrectly estimated the true levels of each of the outcomes. It is possible that we had lower participation from those with lower access to electronic devices or digital literacy, which is more frequent among those from lower socio-economic background. Alternate forms of paper-based data collection, however, were not practical for speed and infection control reasons, and all participating schools routinely communicated with pupils and their parents using electronic communication, which may have mitigated this problem. SIS was an open cohort with participants recruited up to round 5 and some skipping a testing round. The longitudinal patterns and comparisons presented here did not explicitly adjust for demographic changes in participants between rounds, but comparison of characteristics between rounds identified no change in the distribution of key demographic factors.

Testing of antibodies amongst children using oral fluid sampling was non-invasive, which benefited participation and retention in the study, but the assay we deployed is less sensitive than serological sampling. Although we adjusted for this in our analysis, anti-NP IgG may wane at a faster rate than spike protein antibodies.^17^ Thus, we may have underestimated antibody prevalence in children in this study. We were surprised to see stable or perhaps even falling antibody prevalence among students in the summer term in our study. A further limitation is that our study did not explicitly study risks of re-infection or antibody waning, phenomena that were thought to be relatively rare at the time of the design and implementation of SIS.^28^ Further, there is a risk of bias in our comparison of the CIS and SIS in the North-West region due to differences in study samples, age-groups, sampling methods and the timing of our estimates, especially at times when the epidemic may have been changing quickly. Our study may have lacked power to test hypotheses for some of the comparisons we show, while selection biases and residual confounding may have affected the validity of some of these comparisons.

The COVID-19 pandemic has changed rapidly, including the emergence of new variants over time, and changing guidelines and implementation of control measures in schools. This research was undertaken in England from November 2020 to July 2021 during a period when schools were implementing a wide range of challenging measures to reduce coronavirus transmission.^29^ Many of these measures, e.g., isolation of all “bubble” contacts if one person was infected within the bubble, are no longer recommended in English schools.^30^ Hence, conclusions based on this research may not be applicable in the current context. Further, in making comparisons with regional rates among children, our analyses were restricted to the North-West region as we included more schools from this area, and hence may not represent national patterns. The research also preceded the arrival of the Omicron variant, which is more easily transmitted than previous variants.^31^^,^^32^ Further research is needed to assess coronavirus transmission in educational settings in this rapidly changing context.

This study contributes important primary data to the evidence on SARS-CoV-2 infection among students, school staff, and within school settings. Previous reviews concluded that there was limited high quality evidence available to quantify the extent of transmission in schools.^33^^,^^34^ Early evidence suggested lower risks of susceptibility to infection among younger children compared to older children and adults.^35^ Broadly, our findings are consistent with previous syntheses of relevant evidence. Modeling studies have suggested the potential importance of school-based transmission of SARS-CoV-2.^36^^,^^37^ However, both the World Health Organization and the European Centres for Disease Control synthesized the evidence during 2021 and concluded that, where mitigations are in place, there is limited evidence for rapid transmission of SARS-COV-2 in schools.^38^^,^^39^ Studies in Germany,^40^ Norway,^41^ Australia,^42^ the UK,^43^^,^^44^ and the USA^45^^,^^46^ have all concluded that, with mitigations in place, the overall risk of transmission in schools was limited. A systematic review of the risk of transmission involving school staff found mixed evidence,^47^ while other evidence from England suggests similar infection (ONS-CIS) and antibody rates (SIS) compared to working age adults.^29^ A study in Italy also found antibody rates rise in teachers over the school year but concluded that school opening did not “amplify transmission”.^48^ Attack rates involving staff and students in schools are highly variable across studies, but a recent meta-analysis of five contact-tracing studies found a 10-fold lower attack rate (0.7% of contacts becoming infected) in schools than households (7.6%).^49^

The role of school closures in responding to the pandemic of SARS-CoV-2 has been highly contested.^50^ The need to minimize transmission in schools, as well as to prevent loss of learning, remains of paramount importance especially in the context of low vaccinations in school-age children and/or with mRNA vaccines in children providing limited short-term protection against infection and transmission. School closures have wide implications for children and their families, including adverse educational,^51^^,^^52^ economic and well-being effects, with concerns about exacerbating existing inequities.^53^^,^^54^ School closures are, therefore, widely considered a measure of last resort relative to mitigation measures aiming to minimise closures. A systematic review of the impact of school closures on transmission reported high levels of bias and potential confounding in the evidence base, but some evidence that school reopening at time of low incidence was not associated with subsequent increase in community-based transmission.^55^^,^^56^

Two reviews have explored a wide range of evidence on the feasibility, effectiveness and modelled impact of mitigation strategies implemented in schools.^57^^,^^58^ These reviews suggested a range of potential strategies that schools might implement with respect to social distancing within schools, exclusion of symptomatic individuals and other measures with school closures as a last resort owing to their potential negative indirect effects. These strategies were supported by modelling under a range of assumptions. There was a high degree of overlap between guidance issued by different authorities to schools, but little real-world data on the effectiveness of any measures.^59^ An important contribution of our study is our focus on the epidemiology of SARS-CoV-2, with prospective and regular in-school testing, that allowed comparison with background community rates. Taken together, the data also suggest little difference between staff and students in current infection prevalence while at school, and between primary and secondary schools.

Our study addressed urgent questions about the epidemiology of SARS-CoV2 among school populations.^60^ We also aimed to understand how to minimise these risks while keeping schools open.^37^^,^^61^^,^^62^ Our data suggest that schools are a potential location for transmission. Although observational in nature, and subject to a range of limitations, the results we present are consistent with the idea that measures introduced by schools in England may have been partially effective at reducing the risk of on-site infection in schools during the 2020–21 school year. However, evidence also suggests that implementing some of the preventive measures created significant challenges for schools.^63^ Further evaluation is needed to inform ongoing policy in relation both to the ongoing SARS-CoV-2 pandemic, and school preparedness for future infectious disease outbreaks.

## Contributors

JH, SML, SNL, CB, PM, NS, PND, ID, FD and ER were responsible for conceptualisation and study design and methodology. JH, SNL, SML, ID and ER obtained funding. JH, SML, SNL, SI, JP, FD and ER contributed to project administration and provided technical or material support. WEO, JS, KEH, GI, BF, AJ, PND, NS, CB, PM, JH, SML and SNL were involved in acquisition of data. WEO and BF conducted analyses with support from KEH and JS and oversight from EA, JH, and SNL. JS and AJ were responsible for data validation and verification. SML, JH, WEO, KEH, and JS drafted the manuscript. PM, NS, CB, AJ, WJE, CWG, TC, JG, GI, and SNL gave critical revision of the manuscript for important intellectual content. All authors contributed to reviewing and editing of the manuscript. All authors had access to the data. SML and JH had final responsibility to submit for publication.

## Data sharing statement

De-identified study data are available for access by accredited researcher in the ONS Secure Research Service (SRS) for accredited research purposes under part 5, chapter 5 of the Digital Economy Act 2017. For further information about accreditation, contact Research.support@ons.gov.uk or visit the SRS website.

## Ethics approval

The Public Health England Research Support and Governance Office (NR0237) and the London School of Hygiene & Tropical Medicine Ethics Review Committee (ref: 22657).

## Provenance and peer review

Not commissioned; peer reviewed for ethical and funding approval prior to submission.

## Declaration of interests

None declared.

## Appendix Supplementary materials

Image, application 1
