# Supplementary material for: Epidemiology of SARS-CoV-2 infection among staff and students in a cohort of English primary and secondary schools during 2020–2021
Source: Lancet Reg Health Eur. 2022 Aug 24;21:100471. doi: 10.1016/j.lanepe.2022.100471 (PMC9398464; doi:10.1016/j.lanepe.2022.100471)
Supplement: Supplementary file 1 [file mmc1.docx]

**Supplementary Information**

Contents

[Figure S1 – Flowchart describing eligibility, enrolment, withdrawal, participation and response rates for primary schools, primary-school staff, primary-school students over 6 SIS rounds, 2020-2021 2](#_Toc99960516)

[Figure S2 – Flowchart describing eligibility, enrolment, withdrawal, participation and response rates for secondary schools, secondary school staff and secondary school students over 6 SIS rounds, 2020-2021 4](#_Toc99960517)

[Table S1a: Sociodemographic characteristics of primary-school staff who participated in SIS rounds 6](#_Toc99960518)

[Table S1b: Sociodemographic characteristics of primary-school students who participated in SIS rounds 7](#_Toc99960519)

[Table S1c: Sociodemographic characteristics of secondary-school staff who participated in SIS rounds 8](#_Toc99960520)

[Table S1d: Sociodemographic characteristics of secondary-school students who participated in SIS rounds 9](#_Toc99960521)

[Table S2 – Current infection prevalence at each round by school and participant type during 6 SIS rounds, 2020-2021, accounting for clustering by school and local area 10](#_Toc99960522)

[Table S3 – Antibody prevalence at each round by school and participant type during 6 SIS rounds, 2020-2021, accounting for clustering by school and local area 11](#_Toc99960523)

[Table S4 – Incidence of antibody acquisition, per 1000 person-weeks, between SIS rounds by school and participant type, 2020-2021, accounting for clustering by school. 12](#_Toc99960524)

[Table S5a – Infection prevalence in schools at each round by Local Authority area – primary-school staff 13](#_Toc99960525)

[Table S5b – Infection prevalence in schools at each round by Local Authority area – primary-school students 14](#_Toc99960526)

[Table S5c – Infection prevalence in schools at each round by Local Authority area – secondary-school staff 15](#_Toc99960527)

[Table S5d – Infection prevalence in schools at each round by Local Authority area – secondary-school students 16](#_Toc99960528)

[Table S6a – Antibody prevalence in schools at each round by Local Authority area – primary-school staff 17](#_Toc99960529)

[Table S6b – Antibody prevalence in schools at each round by Local Authority area – primary-school students 18](#_Toc99960530)

[Table S6c – Antibody prevalence in schools at each round by Local Authority area – secondary-school staff 19](#_Toc99960531)

[Table S6d – Antibody prevalence in schools at each round by Local Authority area – secondary-school students 20](#_Toc99960532)

[Table S7a – Antibody prevalence excluding home tests in schools at each round by Local Authority area – primary-school staff 21](#_Toc99960533)

[Table S7b – Antibody prevalence excluding home tests in schools at each round by Local Authority area – primary-school students 22](#_Toc99960534)

[Table S7c – Antibody prevalence excluding home tests in schools at each round by Local Authority area – secondary-school staff 23](#_Toc99960535)

[Table S7d – Antibody prevalence excluding home tests in schools at each round by Local Authority area – secondary-school students 24](#_Toc99960536)

Figure S1 – Flowchart describing eligibility, enrolment, withdrawal, participation and response rates for primary schools, primary-school staff, primary-school students over 6 SIS rounds, 2020-2021


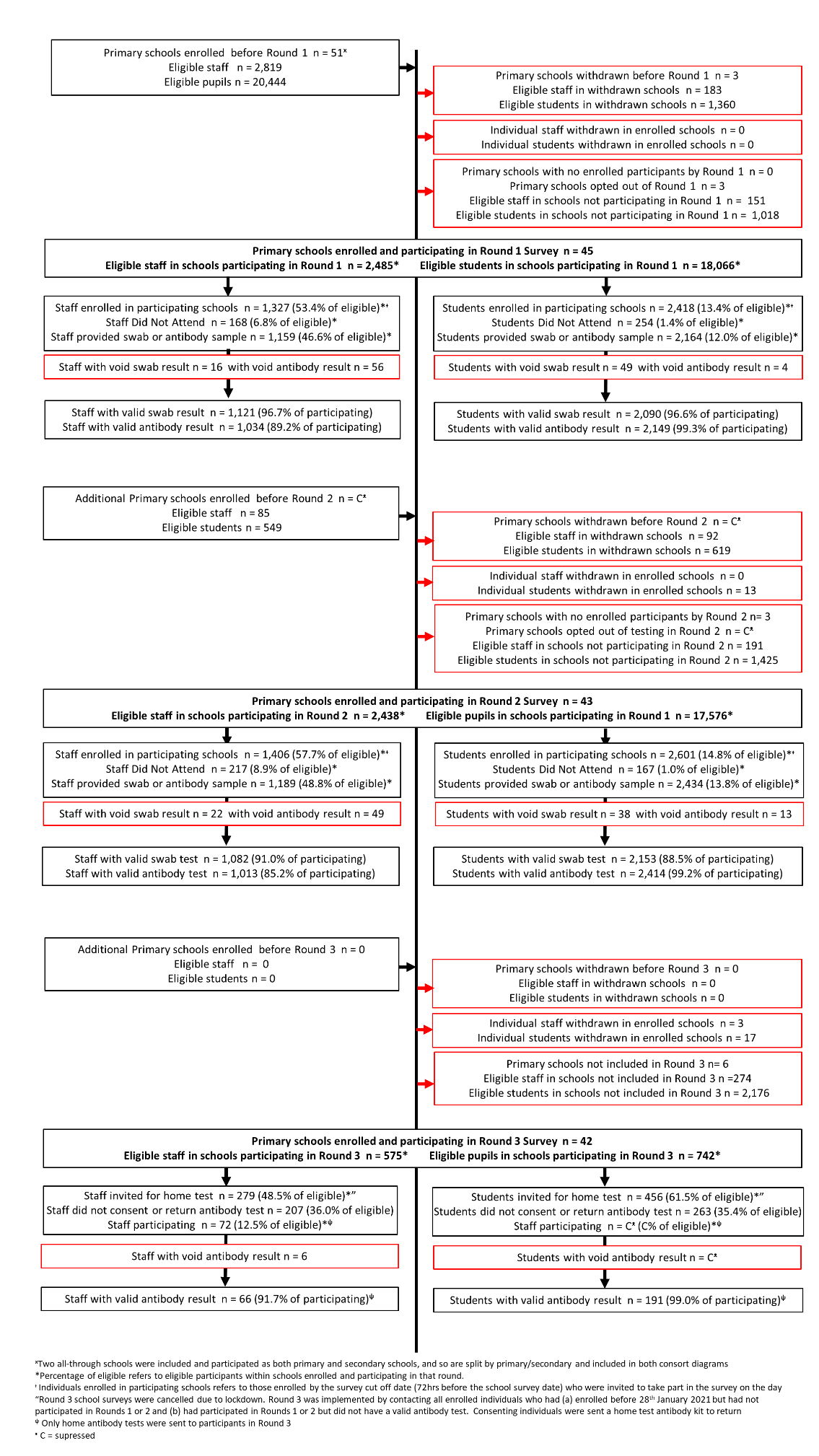


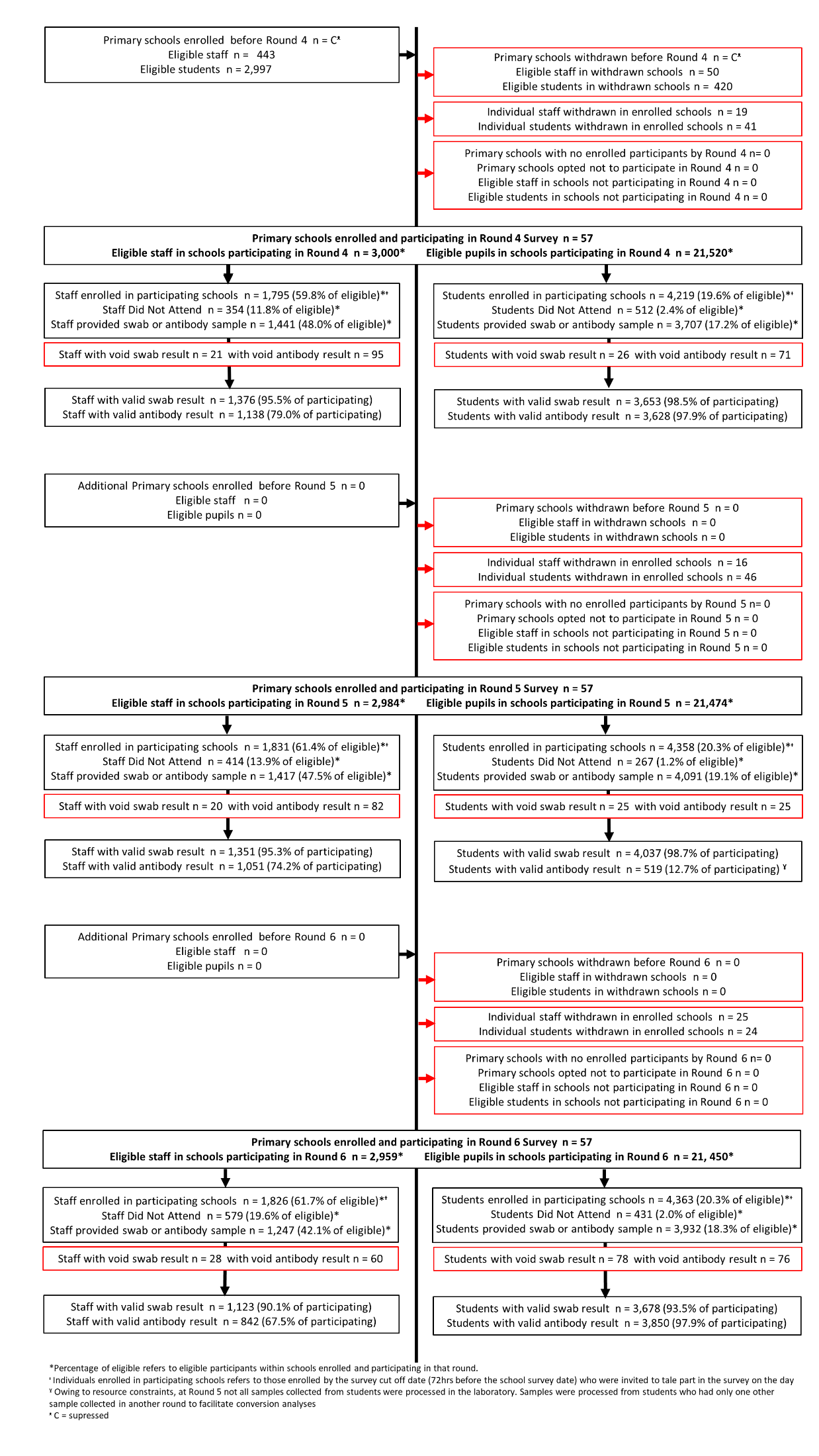


Figure S2 – Flowchart describing eligibility, enrolment, withdrawal, participation and response rates for secondary schools, secondary school staff and secondary school students over 6 SIS rounds, 2020-2021


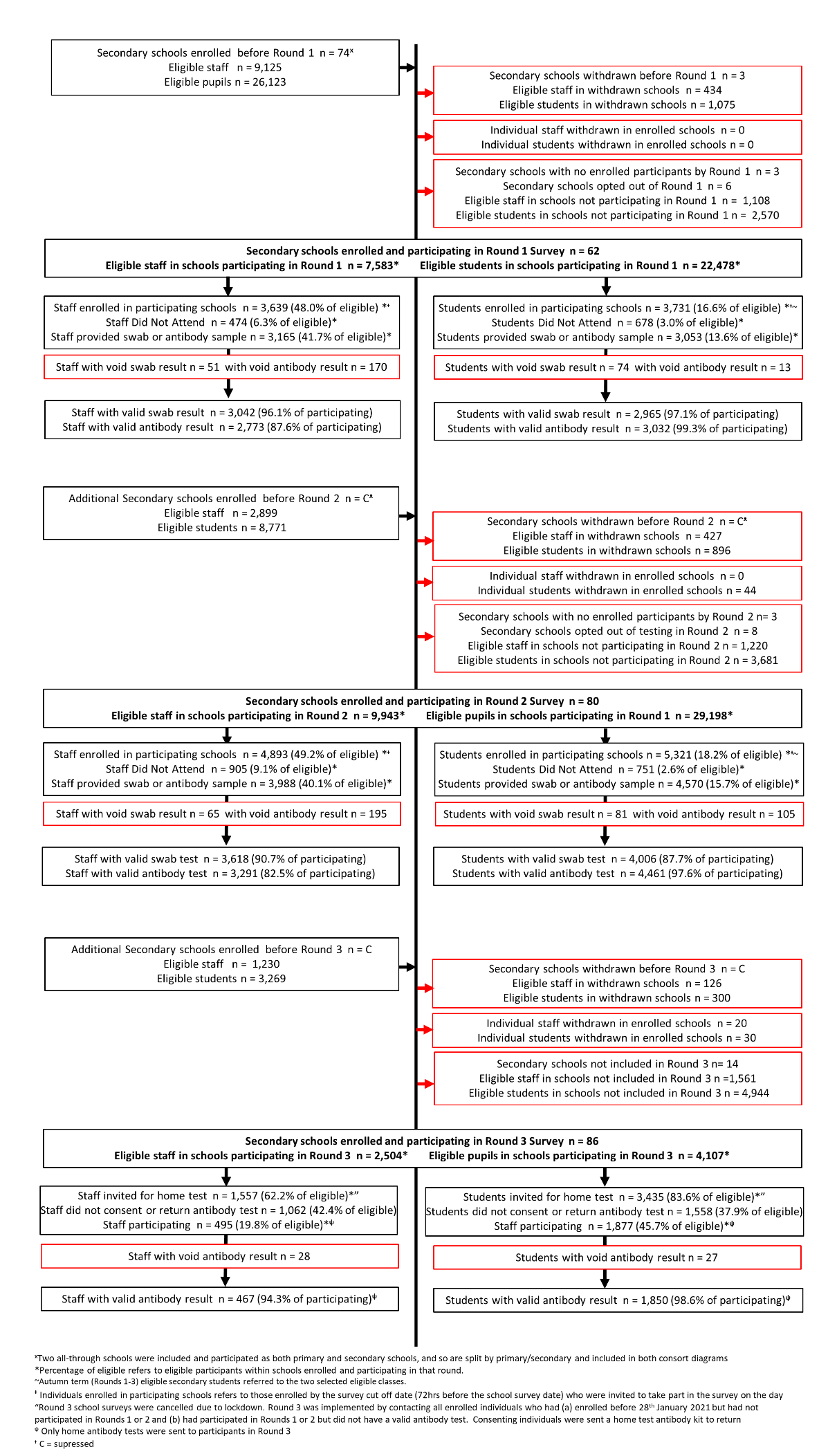


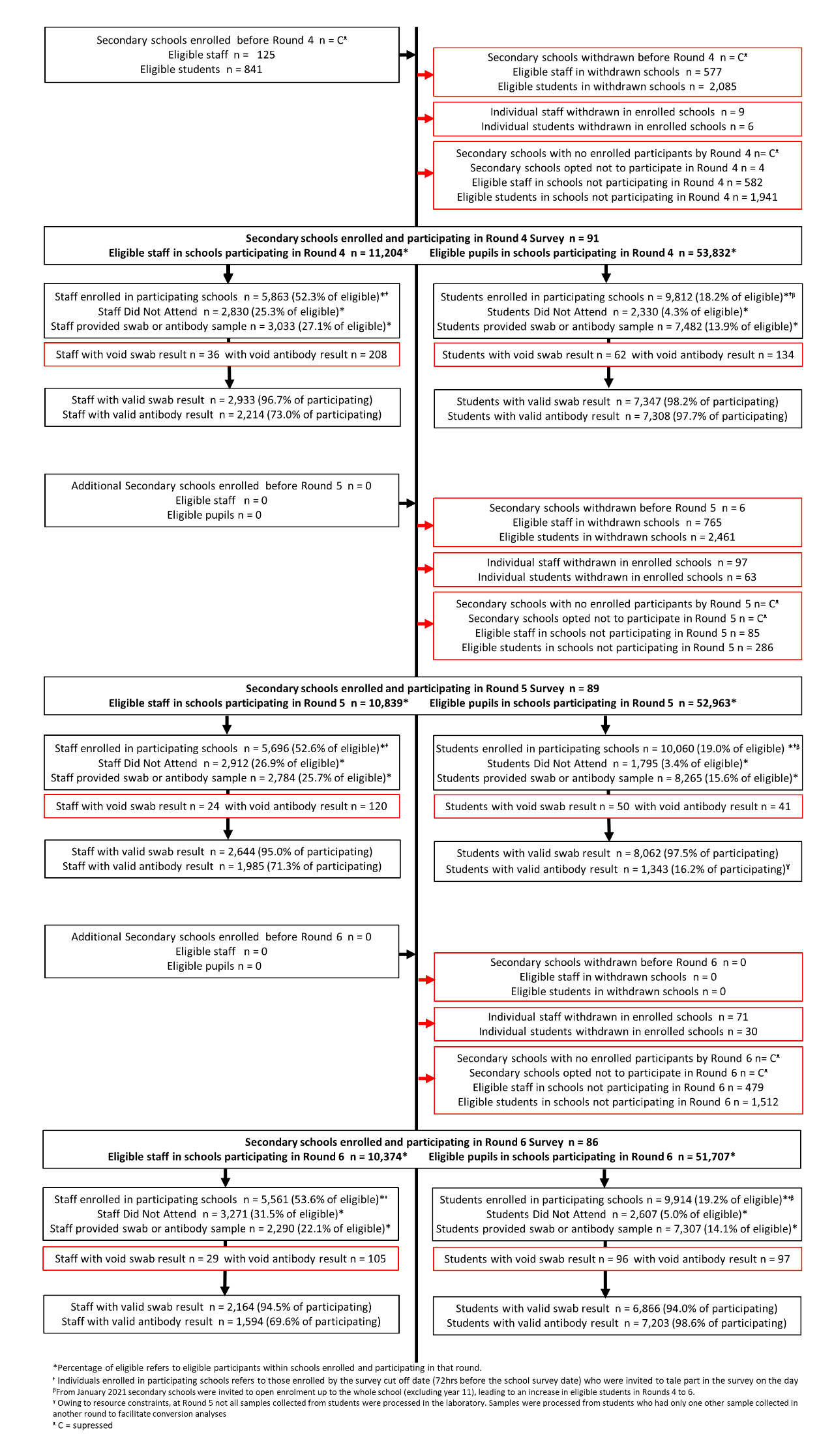


Table S1a: Sociodemographic characteristics of primary-school staff who participated in SIS rounds

|  |  | **Round 1**  **n (%)** | **Round 2**  **n (%)** | **Round 3**  **n (%)** | **Round 4**  **n (%)** | **Round 5**  **n (%)** | **Round 6**  **n (%)** |
| --- | --- | --- | --- | --- | --- | --- | --- |
| **Overall** | -- | 1159 (100.0) | 1189 (100.0) | 72 (100.0) | 1441 (100.0) | 1417 (100.0) | 1247 (100.0) |
| **Transmission Sep-2020** | Low | 392 (33.8) | 395 (33.2) | 34 (47.2) | 454 (31.5) | 435 (30.7) | 393 (31.5) |
|  | High | 767 (66.2) | 794 (66.8) | 38 (52.8) | 987 (68.5) | 982 (69.3) | 854 (68.5) |
| **School rural/urban** | Rural | 80 (6.9) | 62 (5.2) | 7 (9.7) | 128 (8.9) | 112 (7.9) | 113 (9.1) |
|  | Urban city and town | 360 (31.1) | 404 (34.0) | 34 (47.2) | 556 (38.6) | 554 (39.1) | 500 (40.1) |
|  | Urban conurbation | 719 (62.0) | 723 (60.8) | 31 (43.1) | 757 (52.5) | 751 (53.0) | 634 (50.8) |
| **Age group (years)** | <5 years | 0 (0.0) | 0 (0.0) | 0 (0.0) | 0 (0.0) | 0 (0.0) | 0 (0.0) |
|  | 5-9 years | 0 (0.0) | 0 (0.0) | 0 (0.0) | 0 (0.0) | 0 (0.0) | 0 (0.0) |
|  | 10-14 years | 0 (0.0) | 0 (0.0) | 0 (0.0) | 0 (0.0) | 0 (0.0) | 0 (0.0) |
|  | 15+ years | 0 (0.0) | 0 (0.0) | 0 (0.0) | 0 (0.0) | 0 (0.0) | 0 (0.0) |
|  | <35 years | 324 (28.4) | 323 (27.5) | 15 (20.8) | 372 (25.8) | 357 (25.2) | 298 (23.9) |
|  | 35-44 years | 288 (25.2) | 300 (25.5) | 22 (30.6) | 388 (26.9) | 384 (27.1) | 336 (27.0) |
|  | 45-54 years | 330 (28.9) | 342 (29.1) | 21 (29.2) | 439 (30.5) | 442 (31.2) | 412 (33.1) |
|  | 55+ years | 200 (17.5) | 210 (17.9) | 14 (19.4) | C | 234 (16.5) | C |
|  | Data not available | 17 | 14 | 0 | C | 0 | C |
| **Gender** | Male | 109 (9.6) | 123 (10.5) | 5 (6.9) | 144 (10.0) | C | C |
|  | Female | 1032 (90.4) | 1051 (89.5) | 67 (93.1) | 1294 (90.0) | 1267 (89.5) | 1127 (90.6) |
|  | Data not available | 18 | 15 | 0 | 3 | C | C |
| **Ethnicity** | Asian / Asian British | 48 (4.2) | 62 (5.3) | C | 72 (5.0) | 70 (5.0) | 67 (5.4) |
|  | Black / African / Caribbean / Black British | 7 (0.6) | 9 (0.8) | 0 (0.0) | 7 (0.5) | 7 (0.5) | 6 (0.5) |
|  | Mixed / Multiple ethnic groups | 13 (1.1) | 14 (1.2) | C | 12 (0.8) | 14 (1.0) | 10 (0.8) |
|  | Other ethnic group | 4 (0.4) | 4 (0.3) | 0 (0.0) | 5 (0.3) | 5 (0.4) | 5 (0.4) |
|  | White | 1067 (93.7) | 1082 (92.4) | 63 (87.5) | 1338 (93.3) | 1314 (93.2) | 1153 (92.9) |
|  | Data not available | 20 | 18 | 0 | 7 | 7 | 6 |
| **IMD 2019 quintiles** | 1 | 281 (24.8) | 286 (24.6) | 16 (22.9) | 326 (22.8) | 330 (23.5) | 287 (23.3) |
|  | 2 | 220 (19.5) | 223 (19.2) | 13 (18.6) | 293 (20.5) | 288 (20.5) | 248 (20.1) |
|  | 3 | 192 (17.0) | 206 (17.7) | 13 (18.6) | 230 (16.1) | 227 (16.2) | 191 (15.5) |
|  | 4 | 213 (18.8) | 219 (18.9) | C | 297 (20.8) | 291 (20.7) | 267 (21.7) |
|  | 5 | 225 (19.9) | 227 (19.6) | 15 (21.4) | 281 (19.7) | 269 (19.1) | 240 (19.5) |
|  | Data not available | 28 | 28 | C | 14 | 12 | 14 |
| **Job group (staff only)** | Senior leader | 110 (9.8) | 121 (10.5) | C | 143 (10.1) | 143 (10.2) | 123 (10.0) |
|  | Middle leader | 78 (6.9) | 81 (7.0) | 3 (4.3) | 97 (6.8) | 88 (6.3) | 75 (6.1) |
|  | Teacher | 313 (27.9) | 315 (27.2) | 18 (25.7) | 370 (26.0) | 363 (25.9) | 302 (24.5) |
|  | TA / Special Ed | 349 (31.1) | 351 (30.4) | 25 (35.7) | 476 (33.5) | 474 (33.9) | 426 (34.6) |
|  | Admin / Pastoral | 115 (10.2) | 112 (9.7) | 9 (12.9) | 144 (10.1) | 153 (10.9) | 142 (11.5) |
|  | Cater/Clean/Maintenance | 78 (6.9) | 91 (7.9) | 6 (8.6) | 94 (6.6) | 88 (6.3) | 83 (6.7) |
|  | Other | 80 (7.1) | 85 (7.4) | 6 (8.6) | 98 (6.9) | 90 (6.4) | 80 (6.5) |
|  | Data not available | 36 | 33 | C | 19 | 18 | 16 |
| **1 or more vaccine doses by round 6** | No | 165 (14.2) | 165 (13.9) | 10 (13.9) | 174 (12.1) | 149 (10.5) | 129 (10.3) |
|  | Yes | 994 (85.8) | 1024 (86.1) | 62 (86.1) | 1267 (87.9) | 1268 (89.5) | 1118 (89.7) |

C = Suppressed

Table S1b: Sociodemographic characteristics of primary-school students who participated in SIS rounds

|  |  | **Round 1**  **n (%)** | **Round 2**  **n (%)** | **Round 3**  **n (%)** | **Round 4**  **n (%)** | **Round 5**  **n (%)** | **Round 6**  **n (%)** |
| --- | --- | --- | --- | --- | --- | --- | --- |
| **Overall** | -- | 2164 (100.0) | 2434 (100.0) | 193 (100.0) | 3707 (100.0) | 4091 (100.0) | 3932 (100.0) |
| **Transmission Sep-2020** | Low | 526 (24.3) | 731 (30.0) | 87 (45.1) | 1282 (34.6) | 1411 (34.5) | 1392 (35.4) |
|  | High | 1638 (75.7) | 1703 (70.0) | 106 (54.9) | 2425 (65.4) | 2680 (65.5) | 2540 (64.6) |
| **School rural/urban** | Rural | 330 (15.2) | 214 (8.8) | 19 (9.8) | 453 (12.2) | 502 (12.3) | 504 (12.8) |
|  | Urban city and town | 700 (32.3) | 938 (38.5) | 99 (51.3) | 1657 (44.7) | 1845 (45.1) | 1766 (44.9) |
|  | Urban conurbation | 1134 (52.4) | 1282 (52.7) | 75 (38.9) | 1597 (43.1) | 1744 (42.6) | 1662 (42.3) |
| **Age group (years)** | <5 years | 192 (9.0) | 219 (9.1) | 13 (6.9) | 287 (7.7) | C | 295 (7.5) |
|  | 5-9 years | 1543 (72.7) | 1739 (72.5) | 136 (72.0) | 2646 (71.4) | 2898 (70.9) | 2779 (70.7) |
|  | 10-14 years | 387 (18.2) | 439 (18.3) | 40 (21.2) | 771 (20.8) | 887 (21.7) | 857 (21.8) |
|  | 15+ years | 0 (0.0) | 0 (0.0) | 0 (0.0) | 0 (0.0) | 0 (0.0) | 0 (0.0) |
|  | <35 years | 0 (0.0) | 0 (0.0) | 0 (0.0) | 0 (0.0) | 0 (0.0) | 0 (0.0) |
|  | 35-44 years | 0 (0.0) | 0 (0.0) | 0 (0.0) | 0 (0.0) | 0 (0.0) | 0 (0.0) |
|  | 45-54 years | 0 (0.0) | 0 (0.0) | 0 (0.0) | 0 (0.0) | 0 (0.0) | 0 (0.0) |
|  | 55+ years | 0 (0.0) | 0 (0.0) | 0 (0.0) | 0 (0.0) | 0 (0.0) | C |
|  | Data not available | 42 | 37 | 4 | 3 | C | C |
| **Gender** | Male | 1098 (51.8) | 1210 (50.6) | C | 1857 (50.2) | 2055 (50.3) | 1975 (50.3) |
|  | Female | 1023 (48.2) | 1183 (49.4) | 97 (51.1) | 1841 (49.8) | 2029 (49.7) | 1950 (49.7) |
|  | Data not available | 43 | 41 | C | 9 | 7 | 7 |
| **Ethnicity** | Asian / Asian British | 186 (8.8) | 267 (11.2) | 29 (15.5) | 420 (11.4) | 471 (11.6) | 482 (12.4) |
|  | Black / African / Caribbean / Black British | 30 (1.4) | 65 (2.7) | C | 93 (2.5) | 108 (2.7) | 101 (2.6) |
|  | Mixed / Multiple ethnic groups | 129 (6.1) | 149 (6.3) | 12 (6.4) | 230 (6.3) | 233 (5.8) | 226 (5.8) |
|  | Other ethnic group | 21 (1.0) | 31 (1.3) | C | 36 (1.0) | 46 (1.1) | 46 (1.2) |
|  | White | 1744 (82.7) | 1869 (78.5) | 140 (74.9) | 2894 (78.8) | 3192 (78.8) | 3034 (78.0) |
|  | Data not available | 54 | 53 | 6 | 34 | 41 | 43 |
| **IMD 2019 quintiles** | 1 | 518 (24.6) | 643 (27.1) | 56 (29.5) | 994 (27.0) | 1138 (28.0) | 1115 (28.6) |
|  | 2 | 421 (20.0) | 509 (21.4) | 26 (13.7) | 774 (21.1) | 877 (21.6) | 839 (21.5) |
|  | 3 | 278 (13.2) | 354 (14.9) | 36 (18.9) | 581 (15.8) | 640 (15.8) | 638 (16.3) |
|  | 4 | 439 (20.8) | 419 (17.7) | C | 663 (18.0) | 721 (17.7) | 674 (17.3) |
|  | 5 | 450 (21.4) | 448 (18.9) | 35 (18.4) | 663 (18.0) | 686 (16.9) | 638 (16.3) |
|  | Data not available | 58 | 61 | C | 32 | 29 | 28 |
| **Key Stage (year group)** | KS-1 | 938 (44.2) | 1061 (44.2) | 87 (46.3) | C | 1621 (39.7) | 1571 (40.0) |
|  | KS-2 | 1185 (55.8) | 1337 (55.8) | 101 (53.7) | 2211 (59.7) | 2465 (60.3) | 2359 (60.0) |
|  | KS-3 | 0 (0.0) | 0 (0.0) | 0 (0.0) | 0 (0.0) | 0 (0.0) | 0 (0.0) |
|  | KS-4 | 0 (0.0) | 0 (0.0) | 0 (0.0) | 0 (0.0) | 0 (0.0) | 0 (0.0) |
|  | KS-5 | 0 (0.0) | 0 (0.0) | 0 (0.0) | 0 (0.0) | 0 (0.0) | C |
|  | Data not available | 41 | 36 | 5 | C | 5 | C |

C = Suppressed

Table S1c: Sociodemographic characteristics of secondary-school staff who participated in SIS rounds

|  |  | **Round 1**  **n (%)** | **Round 2**  **n (%)** | **Round 3**  **n (%)** | **Round 4**  **n (%)** | **Round 5**  **n (%)** | **Round 6**  **n (%)** |
| --- | --- | --- | --- | --- | --- | --- | --- |
| **Overall** | -- | 3165 (100.0) | 3988 (100.0) | 495 (100.0) | 3033 (100.0) | 2784 (100.0) | 2290 (100.0) |
| **Transmission Sep-2020** | Low | 1037 (32.8) | 1490 (37.4) | 192 (38.8) | 1112 (36.7) | 1061 (38.1) | 840 (36.7) |
|  | High | 2128 (67.2) | 2498 (62.6) | 303 (61.2) | 1921 (63.3) | 1723 (61.9) | 1450 (63.3) |
| **School rural/urban** | Rural | 226 (7.1) | 267 (6.7) | 12 (2.4) | 174 (5.7) | 157 (5.6) | 136 (5.9) |
|  | Urban city and town | 1542 (48.7) | 1915 (48.0) | 268 (54.1) | 1489 (49.1) | 1375 (49.4) | 1095 (47.8) |
|  | Urban conurbation | 1397 (44.1) | 1806 (45.3) | 215 (43.4) | 1370 (45.2) | 1252 (45.0) | 1059 (46.2) |
| **Age group (years)** | <5 years | 0 (0.0) | 0 (0.0) | 0 (0.0) | 0 (0.0) | C | 0 (0.0) |
|  | 5-9 years | 0 (0.0) | 0 (0.0) | 0 (0.0) | 0 (0.0) | 0 (0.0) | 0 (0.0) |
|  | 10-14 years | 0 (0.0) | 0 (0.0) | 0 (0.0) | 0 (0.0) | 0 (0.0) | 0 (0.0) |
|  | 15+ years | 0 (0.0) | 0 (0.0) | 0 (0.0) | 0 (0.0) | 0 (0.0) | 0 (0.0) |
|  | <35 years | 987 (31.7) | 1247 (31.6) | 151 (30.5) | 931 (30.7) | 828 (29.8) | 627 (27.4) |
|  | 35-44 years | 919 (29.5) | 1177 (29.9) | 140 (28.3) | 932 (30.7) | 805 (28.9) | 694 (30.3) |
|  | 45-54 years | 749 (24.1) | 966 (24.5) | 121 (24.4) | 724 (23.9) | 724 (26.0) | 623 (27.2) |
|  | 55+ years | 455 (14.6) | 552 (14.0) | 83 (16.8) | C | 425 (15.3) | 346 (15.1) |
|  | Data not available | 55 | 46 | 0 | C | C | 0 |
| **Gender** | Male | 847 (27.3) | 1045 (26.5) | C | 758 (25.0) | C | C |
|  | Female | 2258 (72.7) | 2892 (73.5) | 375 (76.1) | 2268 (75.0) | 2084 (75.0) | 1737 (75.9) |
|  | Data not available | 60 | 51 | C | 7 | C | C |
| **Ethnicity** | Asian / Asian British | 115 (3.7) | 164 (4.2) | C | 107 (3.5) | 80 (2.9) | 68 (3.0) |
|  | Black / African / Caribbean / Black British | 24 (0.8) | 35 (0.9) | C | 26 (0.9) | 28 (1.0) | 25 (1.1) |
|  | Mixed / Multiple ethnic groups | 63 (2.0) | 69 (1.8) | C | 66 (2.2) | 57 (2.1) | 49 (2.1) |
|  | Other ethnic group | 17 (0.5) | 21 (0.5) | C | 17 (0.6) | 18 (0.7) | 13 (0.6) |
|  | White | 2878 (92.9) | 3631 (92.6) | 465 (94.7) | 2800 (92.8) | 2583 (93.4) | 2125 (93.2) |
|  | Data not available | 68 | 68 | 4 | 17 | 18 | 10 |
| **IMD 2019 quintiles** | 1 | 460 (15.0) | 617 (15.8) | 64 (13.1) | 493 (16.5) | 434 (15.7) | 365 (16.1) |
|  | 2 | 649 (21.1) | 833 (21.4) | 98 (20.1) | 639 (21.3) | 607 (22.0) | 507 (22.4) |
|  | 3 | 588 (19.1) | 759 (19.5) | 88 (18.1) | 552 (18.4) | 502 (18.2) | 415 (18.3) |
|  | 4 | 697 (22.7) | 853 (21.9) | 103 (21.1) | 655 (21.9) | 621 (22.5) | 484 (21.4) |
|  | 5 | 681 (22.1) | 839 (21.5) | 134 (27.5) | 657 (21.9) | 592 (21.5) | 494 (21.8) |
|  | Data not available | 90 | 87 | 8 | 37 | 28 | 25 |
| **Job group (staff only)** | Senior leader | 257 (8.3) | 308 (7.9) | C | 260 (8.6) | 240 (8.6) | 188 (8.2) |
|  | Middle leader | 677 (21.9) | 820 (20.9) | 88 (17.8) | 598 (19.8) | 525 (18.9) | 450 (19.7) |
|  | Teacher | 1087 (35.1) | 1409 (35.9) | 155 (31.4) | 1022 (33.8) | 902 (32.5) | 777 (34.1) |
|  | TA / Special Ed | 285 (9.2) | 331 (8.4) | 44 (8.9) | 273 (9.0) | 260 (9.4) | 204 (8.9) |
|  | Admin / Pastoral | 475 (15.3) | 633 (16.1) | 104 (21.1) | 522 (17.3) | 546 (19.7) | 421 (18.5) |
|  | Cater/Clean/Maintenance | 104 (3.4) | 146 (3.7) | 31 (6.3) | 113 (3.7) | 88 (3.2) | 74 (3.2) |
|  | Other | 213 (6.9) | 276 (7.0) | 44 (8.9) | 232 (7.7) | 215 (7.7) | 167 (7.3) |
|  | Data not available | 67 | 65 | C | 13 | 8 | 9 |
| **1 or more vaccine doses by round 6** | No | 420 (13.3) | 513 (12.9) | 53 (10.7) | 325 (10.7) | 254 (9.1) | 208 (9.1) |
|  | Yes | 2745 (86.7) | 3475 (87.1) | 442 (89.3) | 2708 (89.3) | 2530 (90.9) | 2082 (90.9) |

C = Suppressed

Table S1d: Sociodemographic characteristics of secondary-school students who participated in SIS rounds

|  |  | **Round 1**  **n (%)** | **Round 2**  **n (%)** | **Round 3**  **n (%)** | **Round 4**  **n (%)** | **Round 5**  **n (%)** | **Round 6**  **n (%)** |
| --- | --- | --- | --- | --- | --- | --- | --- |
| **Overall** | -- | 3053 (100.0) | 4570 (100.0) | 1877 (100.0) | 7482 (100.0) | 8256 (100.0) | 7297 (100.0) |
| **Transmission Sep-2020** | Low | 1207 (39.5) | 1844 (40.4) | 879 (46.8) | 3081 (41.2) | 3250 (39.4) | 2995 (41.0) |
|  | High | 1846 (60.5) | 2726 (59.6) | 998 (53.2) | 4401 (58.8) | 5006 (60.6) | 4302 (59.0) |
| **School rural/urban** | Rural | 276 (9.0) | 395 (8.6) | 30 (1.6) | 337 (4.5) | 368 (4.5) | 378 (5.2) |
|  | Urban city and town | 1539 (50.4) | 2375 (52.0) | 1215 (64.7) | 4676 (62.5) | 5018 (60.8) | 4595 (63.0) |
|  | Urban conurbation | 1238 (40.6) | 1800 (39.4) | 632 (33.7) | 2469 (33.0) | 2870 (34.8) | 2324 (31.8) |
| **Age group (years)** | <5 years | 0 (0.0) | 0 (0.0) | 0 (0.0) | 0 (0.0) | C | 0 (0.0) |
|  | 5-9 years | 0 (0.0) | 0 (0.0) | 0 (0.0) | 0 (0.0) | 0 (0.0) | 0 (0.0) |
|  | 10-14 years | 2558 (84.5) | 3648 (80.2) | 1527 (81.8) | 6335 (84.7) | 7121 (86.3) | 6545 (89.7) |
|  | 15+ years | 470 (15.5) | 898 (19.8) | 340 (18.2) | 1140 (15.3) | 1133 (13.7) | 751 (10.3) |
|  | <35 years | 0 (0.0) | 0 (0.0) | 0 (0.0) | 0 (0.0) | 0 (0.0) | 0 (0.0) |
|  | 35-44 years | 0 (0.0) | 0 (0.0) | 0 (0.0) | 0 (0.0) | 0 (0.0) | 0 (0.0) |
|  | 45-54 years | 0 (0.0) | 0 (0.0) | 0 (0.0) | 0 (0.0) | 0 (0.0) | 0 (0.0) |
|  | 55+ years | 0 (0.0) | 0 (0.0) | 0 (0.0) | 0 (0.0) | 0 (0.0) | C |
|  | Data not available | 25 | 24 | 10 | 7 | C | C |
| **Gender** | Male | 1522 (50.3) | 2187 (48.2) | 929 (49.8) | 3837 (51.4) | 4086 (49.6) | 3648 (50.1) |
|  | Female | 1503 (49.7) | 2353 (51.8) | 938 (50.2) | 3626 (48.6) | 4153 (50.4) | 3632 (49.9) |
|  | Data not available | 28 | 30 | 10 | 19 | 17 | 17 |
| **Ethnicity** | Asian / Asian British | 180 (6.0) | 323 (7.1) | 74 (4.0) | 452 (6.1) | 436 (5.3) | 420 (5.8) |
|  | Black / African / Caribbean / Black British | 74 (2.5) | 104 (2.3) | 24 (1.3) | 145 (2.0) | 145 (1.8) | 130 (1.8) |
|  | Mixed / Multiple ethnic groups | 129 (4.3) | 187 (4.1) | 74 (4.0) | 339 (4.6) | 347 (4.2) | 320 (4.4) |
|  | Other ethnic group | 30 (1.0) | 52 (1.2) | 11 (0.6) | 71 (1.0) | 64 (0.8) | 59 (0.8) |
|  | White | 2601 (86.3) | 3854 (85.3) | 1673 (90.1) | 6422 (86.4) | 7212 (87.9) | 6318 (87.2) |
|  | Data not available | 39 | 50 | 21 | 53 | 52 | 50 |
| **IMD 2019 quintiles** | 1 | 716 (23.9) | 1044 (23.2) | 382 (20.5) | 1629 (22.0) | 1800 (22.0) | 1528 (21.1) |
|  | 2 | 654 (21.8) | 963 (21.4) | 345 (18.5) | 1518 (20.5) | 1657 (20.3) | 1459 (20.2) |
|  | 3 | 488 (16.3) | 803 (17.8) | 308 (16.6) | 1160 (15.7) | 1319 (16.1) | 1200 (16.6) |
|  | 4 | 585 (19.5) | 856 (19.0) | 344 (18.5) | 1436 (19.4) | 1634 (20.0) | 1414 (19.5) |
|  | 5 | 559 (18.6) | 836 (18.6) | 482 (25.9) | 1664 (22.5) | 1769 (21.6) | 1639 (22.6) |
|  | Data not available | 51 | 68 | 16 | 75 | 77 | 57 |
| **Key Stage (year group)** | KS-1 | 0 (0.0) | 0 (0.0) | 0 (0.0) | C | 0 (0.0) | 0 (0.0) |
|  | KS-2 | 0 (0.0) | 0 (0.0) | 0 (0.0) | 0 (0.0) | 0 (0.0) | 0 (0.0) |
|  | KS-3 | 2222 (73.5) | 3225 (71.1) | 1301 (70.1) | 5528 (74.0) | 6210 (75.3) | 5800 (79.5) |
|  | KS-4 | 466 (15.4) | 579 (12.8) | 364 (19.6) | 1279 (17.1) | 1452 (17.6) | 1202 (16.5) |
|  | KS-5 | 337 (11.1) | 734 (16.2) | 191 (10.3) | 665 (8.9) | 585 (7.1) | C |
|  | Data not available | 28 | 32 | 21 | C | 9 | C |

C = Suppressed

Table S2 – Current infection prevalence at each round by school and participant type during 6 SIS rounds, 2020-2021, accounting for clustering by school and local area

| Participant Type | Round | N | Positive | Prevalence |
| --- | --- | --- | --- | --- |
| **Primary Staff** | 1 | 1121 | 8 | 0.71 (0.19, 1.24) |
|  | 2 | 1078 | 12 | 1.11 (0.54, 1.69) |
|  | 4 | 1343 | C | C |
|  | 5 | 1351 | C | C |
|  | 6 | 1123 | C | C |
| **Primary Students** | 1 | 2090 | 19 | 0.91 (0.30, 1.52) |
|  | 2 | 2124 | 24 | 1.13 (0.42, 1.84) |
|  | 4 | 3578 | C | C |
|  | 5 | 4037 | 4 | 0.10 (0.00, 0.21) |
|  | 6 | 3678 | 14 | 0.38 (0.15, 0.61) |
| **Secondary Staff** | 1 | 3042 | 44 | 1.45 (0.87, 2.02) |
|  | 2 | 3593 | 56 | 1.56 (0.87, 2.25) |
|  | 4 | 2670 | 8 | 0.30 (0.03, 0.57) |
|  | 5 | 2643 | C | C |
|  | 6 | 2162 | 5 | 0.23 (0.05, 0.41) |
| **Secondary Students** | 1 | 2964 | 44 | 1.48 (0.82, 2.15) |
|  | 2 | 3902 | 59 | 1.51 (1.03, 1.99) |
|  | 4 | 6958 | 14 | 0.20 (0.06, 0.35) |
|  | 5 | 8049 | 4 | 0.05 (0.00, 0.10) |
|  | 6 | 6863 | 29 | 0.42 (0.16, 0.69) |

C = Suppressed

Table S3 – Antibody prevalence at each round by school and participant type during 6 SIS rounds, 2020-2021, accounting for clustering by school and local area

| Participant Type | Round | N | Positive | Prevalence | Adjusted Prevalence* |
| --- | --- | --- | --- | --- | --- |
| **Primary Staff** | 1 | 1034 | 119 | 11.51 (6.27, 16.74) | -- |
|  | 2 | 1013 | 138 | 13.62 (8.66, 18.58) | -- |
|  | 3 | 66 | 17 | 25.76 (10.59, 40.92) | -- |
|  | 4 | 1138 | 252 | 22.14 (17.07, 27.22) | -- |
|  | 5 | 1051 | 248 | 23.60 (19.00, 28.19) | -- |
|  | 6 | 842 | 221 | 26.25 (20.43, 32.06) | -- |
| **Primary Students** | 1 | 2149 | 112 | 5.21 (3.26, 7.16) | 5.33 (2.86, 7.80) |
|  | 2 | 2414 | 159 | 6.59 (4.03, 9.14) | 7.07 (3.84, 10.31) |
|  | 3 | 191 | 19 | 9.95 (4.26, 15.63) | 11.33 (4.13, 18.52) |
|  | 4 | 3628 | 430 | 11.85 (9.40, 14.31) | 13.74 (10.63, 16.84) |
|  | 5 | 519 | 90 | 17.34 (11.34, 23.34) | 20.68 (13.09, 28.28) |
|  | 6 | 3850 | 388 | 10.08 (7.54, 12.62) | 11.49 (8.27, 14.71) |
| **Secondary Staff** | 1 | 2773 | 312 | 11.25 (7.77, 14.74) | -- |
|  | 2 | 3291 | 428 | 13.01 (9.73, 16.28) | -- |
|  | 3 | 467 | 82 | 17.56 (11.61, 23.51) | -- |
|  | 4 | 2214 | 433 | 19.56 (13.99, 25.13) | -- |
|  | 5 | 1985 | 428 | 21.56 (15.57, 27.55) | -- |
|  | 6 | 1594 | 374 | 23.46 (16.94, 29.99) | -- |
| **Secondary Students** | 1 | 3032 | 247 | 8.15 (4.84, 11.45) | 9.05 (4.86, 13.23) |
|  | 2 | 4461 | 411 | 9.21 (6.39, 12.04) | 10.40 (6.82, 13.97) |
|  | 3 | 1850 | 304 | 16.43 (11.81, 21.05) | 19.53 (13.69, 25.38) |
|  | 4 | 7308 | 969 | 13.26 (10.10, 16.41) | 15.52 (11.52, 19.51) |
|  | 5 | 1343 | 174 | 12.96 (10.40, 15.51) | 15.13 (11.90, 18.37) |
|  | 6 | 7203 | 737 | 10.23 (8.06, 12.41) | 11.69 (8.93, 14.44) |

*Adjusted for sensitivity (0.80) and specificity (0.99), using p=(q+specificity−1)/(sensitivity+specificity−1), where p is the adjusted proportion positive and q is the observed proportion positive.

Table S4 – Incidence of antibody acquisition, per 1000 person-weeks, between SIS rounds by school and participant type, 2020-2021, accounting for clustering by school.

| Participant Type | Follow-up  Between Rounds | N | Positive | 1000 Person-Weeks | Incidence, per 1000 person-weeks |
| --- | --- | --- | --- | --- | --- |
| Primary Staff | 1 to 2 | 662 | 10 | 2.06 | 4.85 (1.85, 16.60) |
|  | 2 to 3 | C | 0 | 0.01 | C |
|  | 3 to 4 | 23 | C | 0.16 | C |
|  | 2 to 4 | 589 | 68 | 8.76 | 7.76 (5.26, 11.96) |
|  | 4 to 5 | 618 | 8 | 4.91 | 1.63 (0.81, 3.79) |
|  | 5 to 6 | 478 | 6 | 2.67 | 2.25 (0.99, 6.47) |
| Primary Students | 1 to 2 | 1687 | 44 | 5.32 | 8.28 (5.08, 14.20) |
|  | 2 to 3 | 29 | C | 0.23 | C |
|  | 3 to 4 | 148 | 5 | 0.99 | 5.03 (2.27, 13.17) |
|  | 2 to 4 | 1826 | 137 | 27.29 | 5.02 (3.71, 6.91) |
|  | 4 to 5 | 110 | 3 | 0.86 | 3.49 (1.28, 13.73) |
|  | 5 to 6 | 361 | 8 | 1.94 | 4.13 (1.32, 19.10) |
|  | 4 to 6 | 2744 | 47 | 36.87 | 1.27 (0.82, 2.07) |
| Secondary Staff | 1 to 2 | 1659 | 29 | 5.29 | 5.48 (3.83, 8.14) |
|  | 2 to 3 | 10 | 0 | 0.08 | -- |
|  | 3 to 4 | 170 | C | 1.29 | C |
|  | 2 to 4 | 1198 | 90 | 18.21 | 4.94 (3.80, 6.54) |
|  | 4 to 5 | 982 | 11 | 7.15 | 1.54 (0.86, 2.98) |
|  | 5 to 6 | 853 | 6 | 4.88 | 1.23 (0.60, 2.98) |
| Secondary Students | 1 to 2 | 2292 | 58 | 7.23 | 8.02 (6.18, 10.45) |
|  | 2 to 3 | 45 | 4 | 0.35 | 11.40 (3.93, 52.41) |
|  | 3 to 4 | 1164 | 16 | 8.92 | 1.79 (0.99, 3.38) |
|  | 2 to 4 | 2967 | 205 | 45.37 | 4.52 (3.68, 5.57) |
|  | 4 to 5 | 385 | 5 | 2.75 | 1.82 (0.88, 4.48) |
|  | 5 to 6 | 714 | 14 | 4.17 | 3.36 (1.92, 6.42) |
|  | 4 to 6 | 4686 | 68 | 60.51 | 1.12 (0.85, 1.50) |

C = Suppressed

Table S5a – Infection prevalence in schools at each round by Local Authority area – primary-school staff

|  | **Schools in SIS** | **Round 1** |  |  | **Round 2** |  |  | **Round 3** |  |  | **Round 4** |  |  | **Round 5** |  |  | **Round 6** |  |
| --- | --- | --- | --- | --- | --- | --- | --- | --- | --- | --- | --- | --- | --- | --- | --- | --- | --- | --- |
| **Local Authority** | **n** | **mean (sd)** | **range** | **n** | **mean (sd)** | **range** | **n** | **mean (sd)** | **range** | **n** | **mean (sd)** | **range** | **n** | **mean (sd)** | **range** | **n** | **mean (sd)** | **range** |
| Barking and Dagenham | 4 | 0.00 (0.00) | 0.00-0.00 | 4 | 1.11 (2.22) | 0.00-4.44 | 0 | -- | -- | 4 | 0.00 (0.00) | 0.00-0.00 | 4 | 0.00 (0.00) | 0.00-0.00 | 4 | 0.00 (0.00) | 0.00-0.00 |
| Bournemouth, Christchurch and Poole | C | C | C | C | C | C | 0 | -- | -- | 3 | 0.00 (0.00) | 0.00-0.00 | 3 | 0.00 (0.00) | 0.00-0.00 | 3 | 0.00 (0.00) | 0.00-0.00 |
| Bradford | 0 | -- | -- | 0 | -- | -- | 0 | -- | -- | 0 | -- | -- | 0 | -- | -- | 0 | -- | -- |
| Gateshead | 6 | 0.88 (2.15) | 0.00-5.26 | 3 | 0.00 (0.00) | 0.00-0.00 | 0 | -- | -- | 7 | 0.00 (0.00) | 0.00-0.00 | 7 | 0.45 (1.18) | 0.00-3.13 | 7 | 0.00 (0.00) | 0.00-0.00 |
| Knowsley | 5 | 0.00 (0.00) | 0.00-0.00 | 5 | 0.00 (0.00) | 0.00-0.00 | 0 | -- | -- | 5 | 0.00 (0.00) | 0.00-0.00 | 5 | 0.00 (0.00) | 0.00-0.00 | 5 | 0.00 (0.00) | 0.00-0.00 |
| Lancashire | 0 | -- | -- | 0 | -- | -- | 0 | -- | -- | 3 | 0.00 (0.00) | 0.00-0.00 | 3 | 0.98 (1.70) | 0.00-2.94 | 3 | 0.74 (1.28) | 0.00-2.22 |
| Leicester | C | C | C | 3 | 0.00 (0.00) | 0.00-0.00 | 0 | -- | -- | 4 | 1.39 (2.78) | 0.00-5.56 | 4 | 0.00 (0.00) | 0.00-0.00 | 4 | 0.00 (0.00) | 0.00-0.00 |
| Liverpool | 3 | 2.22 (3.85) | 0.00-6.67 | 3 | 1.45 (2.51) | 0.00-4.35 | 0 | -- | -- | C | C | C | C | C | C | C | C | C |
| Manchester | 4 | 0.81 (1.61) | 0.00-3.23 | 4 | 1.25 (1.60) | 0.00-3.33 | 0 | -- | -- | 4 | 0.00 (0.00) | 0.00-0.00 | 4 | 0.00 (0.00) | 0.00-0.00 | 4 | 0.00 (0.00) | 0.00-0.00 |
| Norfolk | 0 | -- | -- | 0 | -- | -- | 0 | -- | -- | 3 | 0.00 (0.00) | 0.00-0.00 | 3 | 0.00 (0.00) | 0.00-0.00 | 3 | 0.00 (0.00) | 0.00-0.00 |
| Reading | 4 | 0.00 (0.00) | 0.00-0.00 | 5 | 2.86 (6.39) | 0.00-14.29 | 0 | -- | -- | 5 | 0.00 (0.00) | 0.00-0.00 | 5 | 0.00 (0.00) | 0.00-0.00 | 5 | 0.00 (0.00) | 0.00-0.00 |
| Redcar and Cleveland | C | C | C | C | C | C | 0 | -- | -- | 4 | 0.00 (0.00) | 0.00-0.00 | 4 | 0.00 (0.00) | 0.00-0.00 | 4 | 1.09 (2.17) | 0.00-4.35 |
| Salford | 6 | 0.88 (2.15) | 0.00-5.26 | 5 | 0.00 (0.00) | 0.00-0.00 | 0 | -- | -- | 6 | 0.00 (0.00) | 0.00-0.00 | 6 | 0.00 (0.00) | 0.00-0.00 | 6 | 0.00 (0.00) | 0.00-0.00 |
| Sunderland | C | C | C | C | C | C | 0 | -- | -- | 4 | 0.00 (0.00) | 0.00-0.00 | 4 | 0.00 (0.00) | 0.00-0.00 | 4 | 0.00 (0.00) | 0.00-0.00 |
| Warrington | 4 | 2.00 (4.00) | 0.00-8.00 | 3 | 1.11 (1.92) | 0.00-3.33 | 0 | -- | -- | 3 | 0.00 (0.00) | 0.00-0.00 | 3 | 0.00 (0.00) | 0.00-0.00 | 3 | 0.00 (0.00) | 0.00-0.00 |

C = Suppressed

Table S5b – Infection prevalence in schools at each round by Local Authority area – primary-school students

|  |  | **Round 1** |  |  | **Round 2** |  |  | **Round 3** |  |  | **Round 4** |  |  | **Round 5** |  |  | **Round 6** |  |
| --- | --- | --- | --- | --- | --- | --- | --- | --- | --- | --- | --- | --- | --- | --- | --- | --- | --- | --- |
| **Local Authority** | **n** | **mean (sd)** | **range** | **n** | **mean (sd)** | **range** | **n** | **mean (sd)** | **range** | **n** | **mean (sd)** | **range** | **n** | **mean (sd)** | **range** | **n** | **mean (sd)** | **range** |
| Barking and Dagenham | 4 | 0.00 (0.00) | 0.00-0.00 | 4 | 0.00 (0.00) | 0.00-0.00 | 0 | -- | -- | 4 | 0.00 (0.00) | 0.00-0.00 | 4 | 0.00 (0.00) | 0.00-0.00 | 4 | 0.76 (1.52) | 0.00-3.03 |
| Bournemouth, Christchurch and Poole | C | C | C | C | C | C | 0 | -- | -- | 3 | 0.00 (0.00) | 0.00-0.00 | 3 | 0.00 (0.00) | 0.00-0.00 | 3 | 0.00 (0.00) | 0.00-0.00 |
| Bradford | 0 | -- | -- | 0 | -- | -- | 0 | -- | -- | 0 | -- | -- | 0 | -- | -- | 0 | -- | -- |
| Gateshead | 7 | 0.97 (1.32) | 0.00-3.03 | 3 | 0.94 (0.98) | 0.00-1.96 | 0 | -- | -- | 7 | 0.00 (0.00) | 0.00-0.00 | 7 | 0.32 (0.61) | 0.00-1.61 | 7 | 0.28 (0.58) | 0.00-1.54 |
| Knowsley | 5 | 0.69 (1.54) | 0.00-3.45 | 5 | 0.95 (2.13) | 0.00-4.76 | 0 | -- | -- | 5 | 0.00 (0.00) | 0.00-0.00 | 5 | 0.00 (0.00) | 0.00-0.00 | 5 | 1.23 (1.69) | 0.00-3.13 |
| Lancashire | 0 | -- | -- | 0 | -- | -- | 0 | -- | -- | 3 | 0.00 (0.00) | 0.00-0.00 | 3 | 0.00 (0.00) | 0.00-0.00 | 3 | 0.57 (1.00) | 0.00-1.72 |
| Leicester | C | C | C | 3 | 1.99 (1.72) | 0.00-3.03 | 0 | -- | -- | 4 | 0.00 (0.00) | 0.00-0.00 | 4 | 0.00 (0.00) | 0.00-0.00 | 4 | 0.86 (1.72) | 0.00-3.45 |
| Liverpool | 3 | 0.21 (0.37) | 0.00-0.64 | 3 | 0.20 (0.35) | 0.00-0.60 | 0 | -- | -- | C | C | C | C | C | C | C | C | C |
| Manchester | 4 | 0.00 (0.00) | 0.00-0.00 | 4 | 0.48 (0.96) | 0.00-1.92 | 0 | -- | -- | 4 | 1.79 (3.57) | 0.00-7.14 | 4 | 1.56 (3.13) | 0.00-6.25 | 4 | 0.00 (0.00) | 0.00-0.00 |
| Norfolk | 0 | -- | -- | 0 | -- | -- | 0 | -- | -- | 3 | 0.00 (0.00) | 0.00-0.00 | 3 | 0.57 (1.00) | 0.00-1.72 | 3 | 0.00 (0.00) | 0.00-0.00 |
| Reading | 4 | 0.00 (0.00) | 0.00-0.00 | 5 | 1.88 (2.28) | 0.00-5.15 | 0 | -- | -- | 5 | 0.00 (0.00) | 0.00-0.00 | 5 | 0.00 (0.00) | 0.00-0.00 | 5 | 0.00 (0.00) | 0.00-0.00 |
| Redcar and Cleveland | C | C | C | C | C | C | 0 | -- | -- | 4 | 0.00 (0.00) | 0.00-0.00 | 4 | 0.00 (0.00) | 0.00-0.00 | 4 | 0.00 (0.00) | 0.00-0.00 |
| Salford | 6 | 1.19 (2.92) | 0.00-7.14 | 5 | 1.76 (3.95) | 0.00-8.82 | 0 | -- | -- | 6 | 0.00 (0.00) | 0.00-0.00 | 6 | 0.00 (0.00) | 0.00-0.00 | 6 | 0.27 (0.67) | 0.00-1.64 |
| Sunderland | C | C | C | C | C | C | 0 | -- | -- | 4 | 0.00 (0.00) | 0.00-0.00 | 4 | 0.00 (0.00) | 0.00-0.00 | 4 | 0.51 (0.63) | 0.00-1.30 |
| Warrington | 4 | 1.98 (1.94) | 0.00-4.41 | 3 | 0.62 (1.08) | 0.00-1.87 | 0 | -- | -- | 3 | 0.00 (0.00) | 0.00-0.00 | 3 | 0.00 (0.00) | 0.00-0.00 | 3 | 1.93 (1.70) | 0.00-3.23 |

C = Suppressed

Table S5c – Infection prevalence in schools at each round by Local Authority area – secondary-school staff

|  |  | **Round 1** |  |  | **Round 2** |  |  | **Round 3** |  |  | **Round 4** |  |  | **Round 5** |  |  | **Round 6** |  |
| --- | --- | --- | --- | --- | --- | --- | --- | --- | --- | --- | --- | --- | --- | --- | --- | --- | --- | --- |
| **Local Authority** | **n** | **mean (sd)** | **range** | **n** | **mean (sd)** | **range** | **n** | **mean (sd)** | **range** | **n** | **mean (sd)** | **range** | **n** | **mean (sd)** | **range** | **n** | **mean (sd)** | **range** |
| Barking and Dagenham | 4 | 1.59 (1.15) | 0.00-2.70 | 4 | 2.64 (1.99) | 0.00-4.26 | 0 | -- | -- | 4 | 0.61 (1.22) | 0.00-2.44 | 4 | 0.00 (0.00) | 0.00-0.00 | 4 | 0.00 (0.00) | 0.00-0.00 |
| Bournemouth, Christchurch and Poole | 7 | 1.19 (3.15) | 0.00-8.33 | 7 | 0.15 (0.41) | 0.00-1.08 | 0 | -- | -- | 7 | 0.00 (0.00) | 0.00-0.00 | 6 | 0.00 (0.00) | 0.00-0.00 | 7 | 0.00 (0.00) | 0.00-0.00 |
| Bradford | 0 | -- | -- | C | C | C | 0 | -- | -- | C | C | C | C | C | C | C | C | C |
| Gateshead | 3 | 3.09 (2.32) | 1.61-5.77 | C | C | C | 0 | -- | -- | 3 | 0.00 (0.00) | 0.00-0.00 | 3 | 0.00 (0.00) | 0.00-0.00 | 3 | 0.00 (0.00) | 0.00-0.00 |
| Knowsley | 3 | 2.20 (2.40) | 0.00-4.76 | 3 | 2.86 (2.78) | 0.00-5.56 | 0 | -- | -- | C | C | C | 3 | 0.00 (0.00) | 0.00-0.00 | 3 | 0.00 (0.00) | 0.00-0.00 |
| Lancashire | 6 | 0.60 (1.46) | 0.00-3.57 | 6 | 2.41 (3.06) | 0.00-6.38 | 0 | -- | -- | 6 | 0.00 (0.00) | 0.00-0.00 | 9 | 0.43 (1.28) | 0.00-3.85 | 8 | 0.48 (1.36) | 0.00-3.85 |
| Leicester | 6 | 0.48 (0.75) | 0.00-1.59 | 6 | 0.61 (1.02) | 0.00-2.44 | 0 | -- | -- | 5 | 0.00 (0.00) | 0.00-0.00 | 5 | 0.00 (0.00) | 0.00-0.00 | 5 | 0.54 (1.21) | 0.00-2.70 |
| Liverpool | 3 | 1.45 (2.51) | 0.00-4.35 | 5 | 2.00 (2.61) | 0.00-6.38 | 0 | -- | -- | 6 | 0.00 (0.00) | 0.00-0.00 | 7 | 0.00 (0.00) | 0.00-0.00 | 7 | 0.31 (0.82) | 0.00-2.17 |
| Manchester | 3 | 2.08 (3.61) | 0.00-6.25 | 4 | 2.50 (2.90) | 0.00-5.33 | 0 | -- | -- | 7 | 0.85 (1.49) | 0.00-3.57 | 7 | 2.86 (7.56) | 0.00-20.00 | 6 | 0.00 (0.00) | 0.00-0.00 |
| Norfolk | 3 | 1.16 (1.03) | 0.00-1.96 | 7 | 0.68 (0.89) | 0.00-1.89 | 0 | -- | -- | 5 | 0.00 (0.00) | 0.00-0.00 | 6 | 0.00 (0.00) | 0.00-0.00 | 6 | 0.00 (0.00) | 0.00-0.00 |
| Reading | C | C | C | C | C | C | 0 | -- | -- | 4 | 0.00 (0.00) | 0.00-0.00 | 4 | 0.00 (0.00) | 0.00-0.00 | 4 | 0.00 (0.00) | 0.00-0.00 |
| Redcar and Cleveland | 8 | 1.66 (1.82) | 0.00-4.00 | 8 | 1.35 (1.49) | 0.00-3.23 | 0 | -- | -- | 8 | 0.00 (0.00) | 0.00-0.00 | 8 | 0.00 (0.00) | 0.00-0.00 | 8 | 0.69 (1.96) | 0.00-5.56 |
| Salford | 5 | 2.81 (3.34) | 0.00-8.16 | 6 | 2.11 (1.92) | 0.00-5.00 | 0 | -- | -- | 6 | 0.26 (0.65) | 0.00-1.59 | 6 | 0.00 (0.00) | 0.00-0.00 | 5 | 0.00 (0.00) | 0.00-0.00 |
| Sunderland | 4 | 1.37 (1.62) | 0.00-3.19 | 5 | 0.00 (0.00) | 0.00-0.00 | 0 | -- | -- | 5 | 0.00 (0.00) | 0.00-0.00 | 7 | 0.00 (0.00) | 0.00-0.00 | 7 | 0.34 (0.90) | 0.00-2.38 |
| Warrington | 6 | 1.98 (2.98) | 0.00-7.69 | 7 | 0.24 (0.63) | 0.00-1.67 | 0 | -- | -- | 9 | 0.74 (1.57) | 0.00-4.44 | 7 | 0.00 (0.00) | 0.00-0.00 | 9 | 0.00 (0.00) | 0.00-0.00 |

C = Suppressed

Table S5d – Infection prevalence in schools at each round by Local Authority area – secondary-school students

|  |  | **Round 1** |  |  | **Round 2** |  |  | **Round 3** |  |  | **Round 4** |  |  | **Round 5** |  |  | **Round 6** |  |
| --- | --- | --- | --- | --- | --- | --- | --- | --- | --- | --- | --- | --- | --- | --- | --- | --- | --- | --- |
| **Local Authority** | **n** | **mean (sd)** | **range** | **n** | **mean (sd)** | **range** | **n** | **mean (sd)** | **range** | **n** | **mean (sd)** | **range** | **n** | **mean (sd)** | **range** | **n** | **mean (sd)** | **range** |
| Barking and Dagenham | 4 | 0.39 (0.78) | 0.00-1.56 | 4 | 3.91 (2.82) | 0.00-6.67 | 0 | -- | -- | 4 | 0.00 (0.00) | 0.00-0.00 | 4 | 0.00 (0.00) | 0.00-0.00 | 4 | 0.00 (0.00) | 0.00-0.00 |
| Bournemouth, Christchurch and Poole | 7 | 0.25 (0.48) | 0.00-1.27 | 7 | 0.94 (0.95) | 0.00-2.11 | 0 | -- | -- | 8 | 0.06 (0.16) | 0.00-0.46 | 8 | 0.00 (0.00) | 0.00-0.00 | 7 | 0.06 (0.15) | 0.00-0.39 |
| Bradford | 0 | -- | -- | C | C | C | 0 | -- | -- | C | C | C | C | C | C | C | C | C |
| Gateshead | 3 | 1.08 (1.14) | 0.00-2.27 | C | C | C | 0 | -- | -- | 3 | 0.00 (0.00) | 0.00-0.00 | 3 | 0.00 (0.00) | 0.00-0.00 | 3 | 0.37 (0.63) | 0.00-1.10 |
| Knowsley | 3 | 5.99 (3.87) | 2.70-10.26 | 3 | 2.81 (3.17) | 0.00-6.25 | 0 | -- | -- | C | C | C | 3 | 0.00 (0.00) | 0.00-0.00 | 3 | 0.00 (0.00) | 0.00-0.00 |
| Lancashire | 6 | 0.96 (1.57) | 0.00-3.70 | 6 | 0.96 (2.36) | 0.00-5.77 | 0 | -- | -- | 6 | 0.79 (1.25) | 0.00-2.70 | 9 | 0.06 (0.17) | 0.00-0.52 | 8 | 0.60 (0.94) | 0.00-2.53 |
| Leicester | 6 | 1.96 (3.41) | 0.00-8.33 | 6 | 1.84 (3.16) | 0.00-7.69 | 0 | -- | -- | 5 | 0.00 (0.00) | 0.00-0.00 | 5 | 0.00 (0.00) | 0.00-0.00 | 5 | 0.40 (0.89) | 0.00-2.00 |
| Liverpool | 3 | 2.28 (2.53) | 0.00-5.00 | 5 | 1.16 (1.21) | 0.00-2.86 | 0 | -- | -- | 6 | 0.26 (0.64) | 0.00-1.57 | 7 | 0.10 (0.26) | 0.00-0.69 | 6 | 0.00 (0.00) | 0.00-0.00 |
| Manchester | 3 | 1.69 (2.94) | 0.00-5.08 | 5 | 2.48 (4.57) | 0.00-10.53 | 0 | -- | -- | 7 | 0.48 (1.28) | 0.00-3.39 | 7 | 0.00 (0.00) | 0.00-0.00 | 5 | 0.40 (0.89) | 0.00-2.00 |
| Norfolk | 3 | 1.19 (2.06) | 0.00-3.57 | 7 | 1.83 (1.44) | 0.00-3.45 | 0 | -- | -- | 5 | 0.00 (0.00) | 0.00-0.00 | 6 | 0.00 (0.00) | 0.00-0.00 | 6 | 0.06 (0.16) | 0.00-0.39 |
| Reading | C | C | C | C | C | C | 0 | -- | -- | 4 | 0.00 (0.00) | 0.00-0.00 | 4 | 0.00 (0.00) | 0.00-0.00 | 4 | 0.00 (0.00) | 0.00-0.00 |
| Redcar and Cleveland | 7 | 3.35 (4.14) | 0.00-10.53 | 8 | 0.99 (1.70) | 0.00-4.76 | 0 | -- | -- | 8 | 0.00 (0.00) | 0.00-0.00 | 8 | 0.00 (0.00) | 0.00-0.00 | 8 | 0.53 (1.27) | 0.00-3.64 |
| Salford | 5 | 0.85 (1.19) | 0.00-2.50 | 6 | 1.15 (1.93) | 0.00-4.65 | 0 | -- | -- | 6 | 0.00 (0.00) | 0.00-0.00 | 6 | 0.00 (0.00) | 0.00-0.00 | 5 | 0.00 (0.00) | 0.00-0.00 |
| Sunderland | 4 | 2.92 (4.10) | 0.00-8.70 | 5 | 0.82 (1.13) | 0.00-2.27 | 0 | -- | -- | 5 | 0.18 (0.41) | 0.00-0.91 | 7 | 0.00 (0.00) | 0.00-0.00 | 6 | 0.47 (0.73) | 0.00-1.55 |
| Warrington | 6 | 1.25 (1.88) | 0.00-4.76 | 7 | 0.95 (1.97) | 0.00-5.26 | 0 | -- | -- | 9 | 0.20 (0.48) | 0.00-1.42 | 7 | 0.14 (0.38) | 0.00-1.00 | 9 | 0.73 (1.24) | 0.00-2.89 |

C = Suppressed

Table S6a – Antibody prevalence in schools at each round by Local Authority area – primary-school staff

|  | **Schools in SIS** | **Round 1** |  |  | **Round 2** |  |  | **Round 3** |  |  | **Round 4** |  |  | **Round 5** |  |  | **Round 6** |  |
| --- | --- | --- | --- | --- | --- | --- | --- | --- | --- | --- | --- | --- | --- | --- | --- | --- | --- | --- |
| **Local Authority** | **n** | **mean (sd)** | **range** | **n** | **mean (sd)** | **range** | **n** | **mean (sd)** | **range** | **n** | **mean (sd)** | **range** | **n** | **mean (sd)** | **range** | **n** | **mean (sd)** | **range** |
| Barking and Dagenham | 4 | 11.73 (8.50) | 0.00-20.00 | 4 | 14.68 (11.26) | 0.00-27.27 | 3 | 11.11 (19.25) | 0.00-33.33 | 4 | 32.71 (13.74) | 12.50-42.42 | 4 | 27.99 (12.33) | 10.00-36.67 | 4 | 38.78 (18.77) | 14.29-60.00 |
| Bournemouth, Christchurch and Poole | C | C | C | C | C | C | 0 | -- | -- | 3 | 7.12 (2.40) | 4.55-9.30 | 3 | 8.54 (3.85) | 4.35-11.90 | 3 | 7.67 (6.98) | 0.00-13.64 |
| Bradford | 0 | -- | -- | 0 | -- | -- | 0 | -- | -- | 0 | -- | -- | 0 | -- | -- | 0 | -- | -- |
| Gateshead | 6 | 7.66 (3.18) | 4.55-13.51 | 5 | 6.77 (5.20) | 0.00-14.29 | 4 | 12.50 (25.00) | 0.00-50.00 | 7 | 21.22 (16.52) | 5.56-54.55 | 7 | 18.27 (17.94) | 0.00-56.52 | 7 | 22.65 (20.77) | 0.00-63.64 |
| Knowsley | 5 | 18.06 (17.02) | 4.17-47.62 | 5 | 14.62 (16.90) | 0.00-42.86 | 5 | 40.00 (54.77) | 0.00-100.00 | 5 | 18.50 (19.73) | 0.00-52.17 | 5 | 21.69 (16.45) | 11.11-50.00 | 5 | 24.65 (15.47) | 8.33-45.45 |
| Lancashire | 0 | -- | -- | 0 | -- | -- | 0 | -- | -- | 3 | 20.04 (17.90) | 7.14-40.48 | 3 | 21.16 (21.76) | 0.00-43.48 | 3 | 20.80 (21.63) | 0.00-43.18 |
| Leicester | C | C | C | 3 | 9.89 (10.00) | 0.00-20.00 | 3 | 33.33 (57.74) | 0.00-100.00 | 4 | 14.59 (11.03) | 4.55-24.14 | 4 | 17.05 (6.83) | 11.11-26.09 | 4 | 16.58 (14.84) | 5.00-37.50 |
| Liverpool | 3 | 25.56 (17.46) | 10.00-44.44 | 3 | 29.98 (8.14) | 23.53-39.13 | C | C | C | C | C | C | C | C | C | C | C | C |
| Manchester | 4 | 9.27 (8.49) | 1.72-21.05 | 4 | 12.40 (7.30) | 3.85-20.00 | C | C | C | 4 | 21.20 (5.64) | 14.00-27.27 | 4 | 24.37 (11.99) | 12.77-36.00 | 4 | 36.65 (15.05) | 16.13-50.00 |
| Norfolk | 0 | -- | -- | 0 | -- | -- | 0 | -- | -- | 3 | 0.00 (0.00) | 0.00-0.00 | 3 | 4.76 (8.25) | 0.00-14.29 | 3 | 3.70 (6.42) | 0.00-11.11 |
| Reading | 4 | 4.38 (3.38) | 0.00-7.69 | 5 | 7.50 (7.58) | 0.00-20.00 | 4 | 0.00 (0.00) | 0.00-0.00 | 5 | 18.48 (3.18) | 13.33-21.43 | 5 | 20.71 (12.10) | 0.00-30.00 | 5 | 11.90 (9.15) | 0.00-23.08 |
| Redcar and Cleveland | C | C | C | C | C | C | 4 | 32.14 (47.20) | 0.00-100.00 | 4 | 28.13 (38.25) | 0.00-84.62 | 4 | 21.35 (24.32) | 0.00-54.17 | 4 | 24.39 (23.88) | 0.00-57.14 |
| Salford | 6 | 23.99 (19.41) | 5.26-56.52 | 5 | 24.58 (21.63) | 0.00-50.00 | 4 | 54.17 (41.67) | 0.00-100.00 | 6 | 29.70 (13.84) | 12.50-53.33 | 6 | 31.80 (19.03) | 5.26-56.52 | 6 | 33.40 (15.54) | 11.11-54.55 |
| Sunderland | C | C | C | C | C | C | C | C | C | 4 | 14.72 (16.60) | 0.00-37.50 | 4 | 18.66 (6.23) | 12.50-27.27 | 4 | 17.63 (20.20) | 0.00-46.15 |
| Warrington | 4 | 6.00 (7.66) | 0.00-16.00 | 3 | 14.88 (16.20) | 0.00-32.14 | C | C | C | 3 | 27.63 (5.48) | 21.43-31.82 | 3 | 22.95 (9.25) | 13.64-32.14 | 3 | 22.97 (6.25) | 16.67-29.17 |

C = Suppressed

Table S6b – Antibody prevalence in schools at each round by Local Authority area – primary-school students

|  |  | **Round 1** |  |  | **Round 2** |  |  | **Round 3** |  |  | **Round 4** |  |  | **Round 5** |  |  | **Round 6** |  |
| --- | --- | --- | --- | --- | --- | --- | --- | --- | --- | --- | --- | --- | --- | --- | --- | --- | --- | --- |
| **Local Authority** | **n** | **mean (sd)** | **range** | **n** | **mean (sd)** | **range** | **n** | **mean (sd)** | **range** | **n** | **mean (sd)** | **range** | **n** | **mean (sd)** | **range** | **n** | **mean (sd)** | **range** |
| Barking and Dagenham | 4 | 10.10 (6.30) | 4.17-16.67 | 4 | 8.75 (3.07) | 4.55-11.76 | C | C | C | 4 | 21.95 (5.63) | 16.67-27.91 | 4 | 48.11 (38.46) | 9.09-100.00 | 4 | 19.58 (6.55) | 12.12-27.08 |
| Bournemouth, Christchurch and Poole | C | C | C | C | C | C | C | C | C | 3 | 6.30 (1.77) | 5.14-8.33 | 3 | 1.28 (2.22) | 0.00-3.85 | 3 | 4.14 (0.87) | 3.14-4.74 |
| Bradford | 0 | -- | -- | 0 | -- | -- | 0 | -- | -- | 0 | -- | -- | 0 | -- | -- | 0 | -- | -- |
| Gateshead | 7 | 5.32 (6.99) | 0.00-20.00 | 5 | 3.67 (1.47) | 1.56-5.26 | 6 | 11.67 (20.41) | 0.00-50.00 | 7 | 12.01 (9.33) | 0.00-25.37 | 7 | 27.33 (34.75) | 0.00-100.00 | 7 | 11.22 (8.22) | 0.00-25.00 |
| Knowsley | 5 | 8.00 (5.64) | 0.00-15.38 | 5 | 12.62 (8.54) | 0.00-20.69 | 4 | 8.33 (16.67) | 0.00-33.33 | 5 | 15.65 (13.06) | 0.00-31.58 | 5 | 13.33 (21.73) | 0.00-50.00 | 5 | 11.69 (8.64) | 0.00-21.05 |
| Lancashire | 0 | -- | -- | 0 | -- | -- | 0 | -- | -- | 3 | 18.36 (18.16) | 7.87-39.33 | 3 | 15.41 (9.62) | 4.35-21.88 | 3 | 14.33 (16.21) | 4.95-33.05 |
| Leicester | C | C | C | 3 | 14.02 (3.99) | 9.52-17.14 | 3 | 24.44 (21.43) | 0.00-40.00 | 4 | 15.88 (8.17) | 7.69-26.67 | 4 | 9.41 (10.91) | 0.00-20.00 | 4 | 12.13 (3.62) | 8.33-16.28 |
| Liverpool | 3 | 7.37 (2.69) | 4.35-9.52 | 3 | 3.84 (3.54) | 0.00-6.98 | C | C | C | C | C | C | C | C | C | C | C | C |
| Manchester | 4 | 25.11 (24.44) | 2.86-57.14 | 4 | 16.61 (11.39) | 9.38-33.33 | 3 | 66.67 (57.74) | 0.00-100.00 | 4 | 18.36 (5.13) | 12.50-23.08 | C | C | C | 4 | 21.69 (11.04) | 11.76-37.50 |
| Norfolk | 0 | -- | -- | 0 | -- | -- | 0 | -- | -- | 3 | 4.34 (1.30) | 3.13-5.71 | 3 | 1.85 (3.21) | 0.00-5.56 | 3 | 1.65 (1.43) | 0.00-2.56 |
| Reading | 4 | 4.55 (5.31) | 0.00-10.00 | 5 | 1.79 (2.80) | 0.00-6.38 | 3 | 0.00 (0.00) | 0.00-0.00 | 5 | 9.03 (6.45) | 0.00-17.53 | 4 | 4.86 (5.73) | 0.00-11.11 | 5 | 6.38 (3.08) | 2.08-9.26 |
| Redcar and Cleveland | C | C | C | C | C | C | 4 | 8.33 (16.67) | 0.00-33.33 | 4 | 7.76 (1.99) | 5.26-9.76 | 4 | 32.58 (16.31) | 12.50-50.00 | 4 | 12.57 (5.86) | 9.09-21.33 |
| Salford | 6 | 9.42 (7.15) | 2.22-23.08 | 5 | 15.07 (10.04) | 3.85-31.43 | 4 | 19.20 (21.50) | 0.00-50.00 | 6 | 15.66 (6.94) | 5.36-24.24 | 6 | 19.88 (29.52) | 0.00-75.00 | 6 | 13.12 (7.31) | 4.88-22.22 |
| Sunderland | C | C | C | C | C | C | C | C | C | 4 | 10.50 (7.82) | 1.54-20.00 | 4 | 9.45 (11.55) | 0.00-23.53 | 4 | 8.79 (6.26) | 2.94-17.65 |
| Warrington | 4 | 3.79 (2.23) | 1.85-6.67 | 3 | 6.26 (4.89) | 1.79-11.48 | C | C | C | 3 | 8.73 (4.20) | 5.56-13.49 | 3 | 16.67 (28.87) | 0.00-50.00 | 3 | 6.73 (3.76) | 2.47-9.60 |

C = Suppressed

Table S6c – Antibody prevalence in schools at each round by Local Authority area – secondary-school staff

|  |  | **Round 1** |  |  | **Round 2** |  |  | **Round 3** |  |  | **Round 4** |  |  | **Round 5** |  |  | **Round 6** |  |
| --- | --- | --- | --- | --- | --- | --- | --- | --- | --- | --- | --- | --- | --- | --- | --- | --- | --- | --- |
| **Local Authority** | **n** | **mean (sd)** | **range** | **n** | **mean (sd)** | **range** | **n** | **mean (sd)** | **range** | **n** | **mean (sd)** | **range** | **n** | **mean (sd)** | **range** | **n** | **mean (sd)** | **range** |
| Barking and Dagenham | 4 | 18.41 (2.88) | 15.00-22.03 | 4 | 19.69 (7.00) | 13.64-29.33 | 5 | 5.71 (12.78) | 0.00-28.57 | 5 | 29.15 (17.64) | 0.00-46.67 | 5 | 41.86 (16.40) | 22.73-66.67 | 4 | 33.94 (14.73) | 20.00-52.94 |
| Bournemouth, Christchurch and Poole | 7 | 3.92 (4.81) | 0.00-11.11 | 8 | 5.82 (7.95) | 0.00-23.53 | 4 | 10.42 (12.50) | 0.00-25.00 | 7 | 7.12 (7.17) | 0.00-18.52 | 7 | 8.56 (9.23) | 0.00-25.00 | 7 | 4.66 (5.65) | 0.00-15.38 |
| Bradford | 0 | -- | -- | C | C | C | C | C | C | C | C | C | C | C | C | C | C | C |
| Gateshead | 3 | 9.84 (7.54) | 3.92-18.33 | 3 | 12.31 (5.68) | 8.77-18.87 | 3 | 14.44 (17.11) | 0.00-33.33 | 3 | 17.11 (7.23) | 8.89-22.45 | 3 | 20.04 (12.60) | 5.88-30.00 | 3 | 22.49 (14.73) | 11.11-39.13 |
| Knowsley | 3 | 14.72 (10.01) | 4.00-23.81 | 3 | 20.34 (9.33) | 12.28-30.56 | 3 | 91.67 (14.43) | 75.00-100.00 | 3 | 25.75 (23.26) | 0.00-45.24 | 3 | 24.25 (21.58) | 8.70-48.89 | 3 | 30.16 (19.31) | 10.00-48.48 |
| Lancashire | 6 | 10.10 (4.99) | 1.85-15.52 | 6 | 12.32 (7.59) | 1.79-21.21 | 7 | 10.99 (11.17) | 0.00-23.08 | 9 | 20.40 (18.96) | 0.00-60.00 | 9 | 27.65 (19.09) | 5.26-59.09 | 8 | 23.24 (17.35) | 0.00-50.00 |
| Leicester | 6 | 11.08 (4.84) | 5.17-17.86 | 6 | 11.34 (4.96) | 7.14-20.00 | 4 | 8.33 (16.67) | 0.00-33.33 | 5 | 18.80 (14.68) | 5.71-43.75 | 5 | 19.01 (12.39) | 7.69-35.29 | 5 | 24.25 (15.64) | 3.57-42.86 |
| Liverpool | 3 | 9.16 (2.56) | 6.78-11.86 | 7 | 14.35 (5.61) | 7.14-22.22 | 6 | 11.20 (13.79) | 0.00-33.33 | 8 | 37.41 (25.56) | 22.64-100.00 | 7 | 20.39 (11.05) | 0.00-37.50 | 7 | 26.52 (18.54) | 0.00-44.44 |
| Manchester | 3 | 17.84 (6.51) | 13.33-25.30 | 4 | 24.36 (13.06) | 9.76-40.91 | 7 | 22.04 (21.90) | 0.00-60.00 | 7 | 24.19 (16.15) | 0.00-44.00 | 7 | 33.42 (11.65) | 14.29-50.00 | 6 | 41.11 (16.66) | 16.67-60.00 |
| Norfolk | 3 | 1.95 (1.89) | 0.00-3.77 | 7 | 5.12 (4.82) | 0.00-11.11 | 6 | 4.76 (7.38) | 0.00-14.29 | 6 | 6.82 (5.42) | 0.00-13.16 | 6 | 10.00 (7.02) | 2.08-21.88 | 6 | 9.05 (6.89) | 0.00-19.23 |
| Reading | C | C | C | C | C | C | 3 | 38.33 (53.49) | 4.48-100.00 | 4 | 12.20 (11.52) | 0.00-26.67 | 4 | 15.65 (13.86) | 0.00-30.77 | 4 | 15.52 (12.66) | 0.00-26.67 |
| Redcar and Cleveland | 8 | 7.13 (6.01) | 0.00-20.00 | 8 | 9.60 (8.29) | 0.00-22.73 | 7 | 12.50 (21.65) | 0.00-50.00 | 8 | 11.54 (12.07) | 0.00-33.33 | 8 | 19.36 (14.61) | 6.25-50.00 | 8 | 15.50 (13.74) | 0.00-36.84 |
| Salford | 5 | 17.14 (8.41) | 3.33-24.24 | 6 | 20.59 (5.68) | 9.76-25.00 | 5 | 32.38 (33.40) | 0.00-66.67 | 6 | 29.23 (4.21) | 25.00-35.00 | 6 | 31.38 (8.06) | 20.83-41.67 | 6 | 43.56 (11.26) | 25.00-60.00 |
| Sunderland | 4 | 10.40 (7.26) | 0.00-16.88 | 6 | 11.61 (6.15) | 0.00-16.67 | 4 | 8.33 (16.67) | 0.00-33.33 | 7 | 22.57 (14.31) | 5.00-40.00 | 7 | 15.53 (8.48) | 8.33-33.33 | 7 | 17.18 (11.42) | 8.00-41.67 |
| Warrington | 6 | 13.17 (8.93) | 0.00-23.48 | 7 | 10.57 (10.33) | 0.00-30.30 | 4 | 25.38 (21.33) | 0.00-50.00 | 9 | 18.53 (9.98) | 0.00-32.26 | 8 | 19.64 (12.17) | 0.00-33.33 | 9 | 19.20 (12.18) | 0.00-40.00 |

C = Suppressed

Table S6d – Antibody prevalence in schools at each round by Local Authority area – secondary-school students

|  |  | **Round 1** |  |  | **Round 2** |  |  | **Round 3** |  |  | **Round 4** |  |  | **Round 5** |  |  | **Round 6** |  |
| --- | --- | --- | --- | --- | --- | --- | --- | --- | --- | --- | --- | --- | --- | --- | --- | --- | --- | --- |
| **Local Authority** | **n** | **mean (sd)** | **range** | **n** | **mean (sd)** | **range** | **n** | **mean (sd)** | **range** | **n** | **mean (sd)** | **range** | **n** | **mean (sd)** | **range** | **n** | **mean (sd)** | **range** |
| Barking and Dagenham | 4 | 11.87 (5.45) | 4.55-17.65 | 4 | 10.38 (3.31) | 5.75-13.33 | 5 | 23.57 (18.60) | 0.00-42.86 | 5 | 23.07 (9.30) | 12.50-34.62 | 5 | 21.82 (43.88) | 0.00-100.00 | 4 | 16.58 (7.76) | 11.11-27.78 |
| Bournemouth, Christchurch and Poole | 7 | 2.38 (1.80) | 0.00-5.26 | 8 | 3.10 (1.75) | 0.00-5.88 | 8 | 4.11 (4.84) | 0.00-12.50 | 8 | 5.32 (2.61) | 0.00-7.60 | 7 | 4.08 (7.32) | 0.00-20.00 | 7 | 5.06 (2.10) | 1.85-8.74 |
| Bradford | 0 | -- | -- | C | C | C | C | C | C | C | C | C | C | C | C | C | C | C |
| Gateshead | 3 | 7.06 (2.13) | 4.76-8.96 | 3 | 5.63 (6.19) | 0.00-12.26 | 3 | 19.65 (17.02) | 0.00-30.00 | 3 | 12.90 (11.75) | 0.00-22.98 | 3 | 26.10 (10.90) | 15.79-37.50 | 3 | 11.46 (2.25) | 10.00-14.04 |
| Knowsley | 3 | 7.63 (6.83) | 2.50-15.38 | 3 | 14.52 (4.43) | 9.80-18.60 | 3 | 37.41 (14.07) | 22.22-50.00 | 3 | 23.49 (5.76) | 19.05-30.00 | 3 | 11.11 (19.25) | 0.00-33.33 | 3 | 11.40 (3.03) | 8.06-14.00 |
| Lancashire | 6 | 9.11 (6.24) | 1.09-17.65 | 6 | 10.82 (8.67) | 3.45-23.15 | 7 | 16.99 (12.39) | 0.00-27.27 | 10 | 14.99 (11.40) | 0.00-33.33 | 9 | 27.11 (35.44) | 0.00-100.00 | 8 | 14.88 (13.71) | 0.00-40.00 |
| Leicester | 6 | 9.74 (10.26) | 0.00-27.27 | 6 | 19.19 (14.10) | 7.14-38.46 | 3 | 33.33 (57.74) | 0.00-100.00 | 5 | 20.44 (13.71) | 8.18-43.75 | 4 | 22.62 (19.20) | 0.00-42.86 | 5 | 16.49 (8.97) | 6.82-30.77 |
| Liverpool | 3 | 8.58 (4.03) | 5.56-13.16 | 7 | 12.19 (8.68) | 4.44-28.57 | 6 | 12.04 (9.79) | 0.00-22.22 | 8 | 10.69 (7.59) | 0.00-19.15 | 7 | 13.87 (14.96) | 0.00-33.33 | 7 | 13.13 (16.69) | 0.00-50.00 |
| Manchester | 3 | 20.47 (19.30) | 0.00-38.33 | 5 | 20.32 (15.20) | 0.00-39.66 | 6 | 27.80 (19.70) | 0.00-50.00 | 7 | 23.33 (14.12) | 0.00-42.42 | 7 | 28.29 (33.26) | 0.00-100.00 | 6 | 22.90 (11.36) | 8.89-34.43 |
| Norfolk | 3 | 1.11 (0.96) | 0.00-1.75 | 7 | 3.88 (2.81) | 0.00-8.25 | 6 | 8.68 (11.43) | 0.00-27.73 | 6 | 8.89 (8.49) | 3.17-25.76 | 6 | 4.07 (6.47) | 0.00-14.44 | 6 | 5.53 (5.36) | 0.00-13.08 |
| Reading | C | C | C | C | C | C | 4 | 14.02 (10.88) | 2.94-28.57 | 4 | 13.58 (8.43) | 5.45-25.00 | 4 | 6.92 (9.05) | 0.00-19.05 | 4 | 11.42 (7.64) | 2.88-20.83 |
| Redcar and Cleveland | 7 | 7.48 (5.71) | 0.00-16.22 | 8 | 9.45 (5.13) | 0.00-15.38 | 8 | 4.03 (8.14) | 0.00-22.22 | 8 | 9.79 (5.04) | 0.00-15.00 | 8 | 12.48 (9.76) | 0.00-25.00 | 8 | 9.84 (7.79) | 0.00-26.09 |
| Salford | 5 | 9.97 (5.73) | 3.13-17.07 | 6 | 7.13 (6.45) | 0.00-16.28 | 4 | 3.57 (7.14) | 0.00-14.29 | 6 | 12.75 (5.30) | 6.25-19.28 | 6 | 22.82 (18.15) | 0.00-40.00 | 6 | 11.61 (5.83) | 0.00-15.71 |
| Sunderland | 4 | 11.41 (6.56) | 3.28-17.39 | 6 | 11.23 (4.85) | 4.29-15.91 | 6 | 32.31 (34.48) | 7.41-100.00 | 7 | 17.31 (5.13) | 10.00-23.53 | 7 | 13.18 (9.42) | 0.00-27.27 | 7 | 12.07 (5.80) | 3.70-20.00 |
| Warrington | 6 | 10.39 (8.59) | 2.94-24.41 | 7 | 9.59 (5.36) | 5.00-19.73 | 6 | 12.76 (11.72) | 0.00-29.63 | 9 | 9.92 (7.35) | 0.00-25.74 | 8 | 18.13 (21.43) | 0.00-50.00 | 9 | 7.98 (5.34) | 1.82-20.94 |

C = Suppressed
